# Grafting Snake Melon [*Cucumis melo* L. subsp. *melo* Var. *flexuosus* (L.) Naudin] in Organic Farming: Effects on Agronomic Performance; Resistance to Pathogens; Sugar, Acid, and VOC Profiles; and Consumer Acceptance

**DOI:** 10.3389/fpls.2021.613845

**Published:** 2021-02-19

**Authors:** Alejandro Flores-León, Santiago García-Martínez, Vicente González, Ana Garcés-Claver, Raúl Martí, Carmen Julián, Alicia Sifres, Ana Pérez-de-Castro, María José Díez, Carmelo López, María Ferriol, Carmina Gisbert, Juan José Ruiz, Jaime Cebolla-Cornejo, Belén Picó

**Affiliations:** ^1^Instituto de Conservación y Mejora de la Agrodiversidad Valenciana, Universitat Politècnica de València, Valencia, Spain; ^2^Escuela Politécnica Superior de Orihuela, Universidad Miguel Hernández, Orihuela, Spain; ^3^Plant Protection Unit/Instituto Agroalimentario de Aragón-IA2, Centro de Investigación y Tecnología Agroalimentaria de Aragón, Universidad de Zaragoza, Zaragoza, Spain; ^4^Horticulture Unit/Instituto Agroalimentario de Aragón-IA2, Centro de Investigación y Tecnología Agroalimentaria de Aragón, Universidad de Zaragoza, Zaragoza, Spain; ^5^Instituto Agroforestal Mediterráneo, Universitat Politècnica de València, Valencia, Spain

**Keywords:** *flexuosus*, grafting, fruit quality, organic agriculture, soilborne pathogens

## Abstract

The performance of snake melon [*Cucumis melo* var. *flexuosus* (L.)] in organic farming was studied under high biotic and salt stress conditions. Soilborne diseases (mainly caused by *Macrophomina phaseolina* and *Neocosmospora falciformis*), combined with virus incidence [*Watermelon mosaic virus* (WMV), *Zucchini yellow mosaic virus* (ZYMV), and *Tomato leaf curl New Delhi virus* (ToLCNDV)] and *Podosphaera xanthii* attacks, reduced yield by more than 50%. Snake melon susceptibility to *M. phaseolina* and *Monosporascus cannonballus* was proved in pathogenicity tests, while it showed some degree of resistance to *Neocosmospora keratoplastica* and *N. falciformis*. On the contrary, salt stress had a minor impact, although a synergic effect was detected: yield losses caused by biotic stress increased dramatically when combined with salt stress. Under biotic stress, grafting onto the melon F_1_Pat81 and wild *Cucumis* rootstocks consistently reduced plant mortality in different agroecological conditions, with a better performance compared to classic *Cucurbita* commercial hybrids. Yield was even improved under saline conditions in grafted plants. A negative effect was detected, though, on consumer acceptability, especially with the use of *Cucurbita* rootstocks. *Cucumis* F_1_Pat81 rootstock minimized this side effect, which was probably related to changes in the profile of sugars, acids, and volatiles. Grafting affected sugars and organic acid contents, with this effect being more accentuated with the use of *Cucurbita* rootstocks than with *Cucumis*. In fact, the latter had a higher impact on the volatile organic compound profile than on sugar and acid profile, which may have resulted in a lower effect on consumer perception. The use of *Cucumis* rootstocks seems to be a strategy to enable organic farming production of snake melon targeted to high-quality markets in order to promote the cultivation of this neglected crop.

## Introduction

Melon (*Cucumis melo* L.) is a member of the Cucurbitaceae family, which comprises several economically important vegetables, including cucumber, watermelon, squash, pumpkin, and gourds (Pitrat, 2008). It is a highly variable species with a relevant economic importance worldwide (global production 27 Mt) (FAOSTAT, 2018). The top 10 producing countries are in Asia (China, Turkey, India, Kazakhstan, and Iran), Africa (Egypt), Europe (Spain), and America (United States, Guatemala, and Mexico). Spain is the 8th highest melon producer in the world, and the first producer and exporter of the European Union (EuroStat, 2019). *C. melo* has been produced in Spain since Roman times. The Roman author Lucius Junius Moderatus *Columella* already mentioned in his writings the cultivation of a snake-shaped *cohombro*, a *C. melo* belonging to the botanical variety *flexuosus* (Hammer and Gladis, 2014), which produced non-sweet fruits. With the arrival of Islam to the Iberian Peninsula, sweet casaba type melons as well as cucumbers (*Cucumis sativus* L.) were introduced into Europe from Central Asia (Paris et al., 2012a, b). The preference of sweet casaba melon as fruit and cucumber as vegetable displaced and diminished the consumption of *flexuosus*-type melons.

Snake melon is also an ancient crop highly appreciated in other Mediterranean, Asia Minor, North Africa, and Near and Middle East countries, where it is known by different local names such as Armenian cucumber, Fakous, Kakri, Cucumaru, Hiti, or Mekte, among others (Merheb et al., 2020). Its long non-sweet, non-aromatic fruits are collected when immature and are consumed as fresh vegetables or pickled (Pitrat, 2016; Solmaz et al., 2016a). They are part of many traditional recipes, used like cucumbers because of their appearance and taste, and are also used in traditional medicine (Ibrahim, 2017). This is one of the less studied melons, although the genetic diversity of Middle East landraces has started to be known (Dastranji et al., 2017; Abu Zaitoun et al., 2018; Merheb et al., 2020).

Despite the fact that it has been a neglected crop, snake melons are still commonly grown in many Mediterranean, Asian, and African countries. In Spain, they remain under cultivation in eastern coastal regions (Valencia, Alicante, and Murcia), where local farmers have conserved landraces locally known as “alficoz or alficoç.” Some of these landraces are cultivated for self-consumption and for local markets as the short shelf life of the fruits, much shorter than that of the cucumbers, hampers their commercialization in distant markets. However, this crop is threatened by severe genetic erosion and Spanish snake melon landraces are maintained *ex situ* in the Universitat Politècnica de València GeneBank.

There exists a global interest for the recovery of heirloom vegetables. Farmers markets, where consumers have access to locally grown vegetables, have continued to rise in popularity. Consumer demands on traditional products are more focused on those produced under sustainable or organic production systems. Organic farming values the use of plant diversity and may constitute the ideal context to promote the cultivation of snake melon (Reganold and Wachter, 2016). The consumer perception of organic food as providing differentiating benefits related to health, nutritional value, and maximum respect to the environment and animals (Massey et al., 2018) can be the basis to revitalize its demand, encourage its cultivation, and promote *in situ* conservation.

Melon organic farming faces several limiting factors that need to be overcome to be economically sustainable. One of the major limitations is the negative effect on yield and yield stability (Seufert et al., 2012; Schrama et al., 2018). Pests and diseases are main factors causing this loss of productivity, as the use of agrochemicals is very limited. Also, the fact that the local production of this crop is often restrained to marginable lands, where not only biotic but also abiotic stressful conditions occur, enhances the challenge.

One way of controlling diseases would be the use of resistant varieties. However, the alficoz, as many heirloom landraces, has been often neglected in melon breeding programs as the main focus was on sweet melons. Some breeding has centered on yield and earlier production, but no cultivars with introgressed resistances are available (Abdelmohsin et al., 2015). Grafting is an effective approach to deal with soilborne pathogens, increasing yields in stressful environments (Bie et al., 2017). Grafting is used in Cucurbits, mainly in watermelons (about the 90% of the watermelon production in many countries is obtained from grafted plants). Grafting is not so popular in melons due to the lack of appropriated rootstocks. Melon grafting is performed primarily to face soilborne pathogens such as *Fusarium* and *Monosporascus* wilts or nematodes (Kyriacou et al., 2018). Compatibility in rootstock–scion combinations and the lack of negative effect on fruit quality are necessary for the successful performance of grafted plants (Picó et al., 2017; Leonardi et al., 2017).

There are many previous studies that report the use of different rootstocks for sweet melons and describe their impact on their aspect and flavor, which is particularly affected by the sugar/acid content and aroma (Colla et al., 2006; Crinò et al., 2007; Condurso et al., 2012; Verzera et al., 2014). These studies clearly show that grafting can have an impact on the quality profile of sweet melons. Also, the impact of grafting in flavor perception through the accumulation of certain compounds has been reported in watermelons (Guler et al., 2014; Fredes et al., 2017; Tripodi et al., 2020). It seems that these effects would be limited in species harvested before biological maturity such as cucumber (Davis et al., 2008), as it would be the case for snake melon.

Only few studies have analyzed flavor preferences of snake melons and their relationship with metabolite accumulation. A recent study with accessions from the Middle East suggested that crispy and non-hollow fruits are not necessarily tastier, but softer and hollow fruits are seldom associated with very good taste (Omari et al., 2018). They also highlighted the existence of a high diversity of taste perception within types. Grafting snake melon has been studied (Maroto et al., 2003), but not the impact of grafting on the quality of this crop. The objective of this study is to fill the gap in the knowledge of the effect of grafting on snake melon agronomic performance under organic farming. The effect of biotic and abiotic stressful conditions is evaluated, as well as the impact of the different types of rootstocks, melon, wild *Cucumis*, and *Cucurbita* hybrids, on fruit characteristics, sensory perception, and sugar, acid, and volatile accumulation.

## Materials and Methods

### Fields Characteristics

The study was performed in three different fields of the Valencian Community (Eastern Spain) (Supplementary Figure 1). The first was located in Moncada, a small town in the province of Valencia (39°33′26.8″ N, 0°25′06.5″ W), in a field with no previous history of melon cultivation, as it had been a *Citrus* orchard for the previous 20 years (Supplementary Figure 1A). The second field assay was located in La Punta, a suburban area of the city of Valencia (39°26′41.3″ N, 0°21′’14.9″ W), with a long history of melon cultivation (Supplementary Figure 1B). The third field assay was in the province of Alicante, in the Natural Park of Carrizales (38°08′32.8″ N, 0°42′44.7″ W). The cultivation plots of Carrizales used in 2018 and 2019 were cultivated with alfalfa and oat, respectively, for the three previous years, and then, 6 months before the assay, were fallowed (Supplementary Figure 1C). Climate data were obtained from public databases (La Punta: Agencia Estatal de Meteorología^1^; Moncada and Carrizales: Sistema de Información Agroclimática para el Regadío^2^).

### Plant Material

A local snake melon cultivar traditionally known as “Alficoz valenciano” obtained from the GeneBank of the Universitat Politècnica de València (accession number BGV004853) was used for the study. This accession was selected in a previous study considering its good field performance regarding yield and quality and its good consumer acceptance.

In order to analyze the effect of grafting on agronomic performance, fruit quality, and sugar, acid, and volatile profile, five rootstocks were selected and used to graft the snake melon cultivar (Supplementary Figure 2). All graftings were compatible, resulting in fully developed adult plants. Rootstocks included F_1_Pat81, an experimental inter-subspecific cross between *C. melo* subsp. *agrestis* Pat 81, resistant to *Monosporascus cannonballus* (Roig et al., 2012), and *C. melo* subsp. *melo* Piel de sapo, two hybrid rootstocks between wild *Cucumis* species, Fian (hybrid *Cucumis ficifolius* × *Cucumis anguria*), and Fimy (hybrid *C. ficifolius* × *Cucumis myriocarpus*), resistant to different soilborne diseases (Cáceres et al., 2017), and two commercial hybrid *Cucurbita maxima* × *Cucurbita moschata* rootstocks (Shintoza and Cobalt). Non-grafted (NG) snake melon plants were used as controls in all the assays. The grafting technique employed for the experiments was the “Tongue approach grafting” method.

### Crop Management

The experiments were conducted under organic production in the three different fields. The three fields represent three different agro-ecological situations, frequent in melon cultivation. All fields have a clay soil (more clay-loam in Moncada). In the latter field, Moncada, the lack of melon cultivation in the 20 previous years provided unstressed conditions. In La Punta, fungal stress had been reported, due to the accumulation of soilborne pathogens after repetitive melon cultivation. In the natural park of Carrizales, the traditional irrigation system uses water coming from a drainage water channel. This water is characterized by its high electrical conductivity, thus resulting in salt stress conditions.

In 2018, the three fields were cultivated. NG plants were cultivated in the three fields. Additionally, in La Punta and Carrizales, snake melon was also cultivated and grafted onto two rootstocks, the experimental melon hybrid F_1_Pat81 and the commercial *Cucurbita* hybrid Cobalt. A randomized complete block design with four plants per treatment and block was used (four blocks).

In 2019, plants were grown in La Punta and Carrizales. This year, the snake melon was cultivated NG and grafted onto five selected rootstocks, the two used in 2018 (F_1_Pat81 and Cobalt), the two experimental wild *Cucumis* rootstocks Fian and Fimy, and one additional commercial *Cucurbita* hybrid, Shintoza, using the same experimental design.

Snake melon plants were transplanted at the 2–3 true leaf stage, between the end of March and the first week of April in the three cultivation sites, in 2018, and the first week of May in 2019. During both years, in the fields, sweet melon Spanish varieties were also cultivated, both grafted and NG.

In Moncada, plants were transplanted onto ridges with black mulch with a separation of 1.1 m between ridges and 1 m between plants. The field was prepared by subsoiling and sheep manure (1 kg m^–2^) was applied, and afterward, the soil was milled to break down clods of soil and mix the manure. Two applications of azadirachtin with *Equisetum arvense* and diatomaceous earth were performed to control aphids and whiteflies. In La Punta, a black plastic mulch was also used, with the separation of 2 m between ridges and 0.6 m between plants. The only further treatment performed was the weeding of the soil between ridges. In Carrizales, plants were spaced 2 m between ridges and 0.9 m between plants, with an additional 0.5 m between each treatment and using black plastic mulch. The field was prepared by subsoiling and applying sheep manure (3 kg m^–2^), and afterward, the soil was milled to break down clods of soil and mix the manure. After transplanting, the plants were covered with a thermal blanket until they reached the appropriate size. The blanket enabled the control of both temperature and humidity, also acting as a barrier against pests. During the crop cycle in Carrizales, two applications were performed with humic and fulvic acids diluted in the water supply. During fruit ripening, one application through the water supply was done of potassium sulfate. Every 15–20 days, sulfur (15–25 kg ha) and Vibafusan G (15 kg ha^–1^) were applied. Finally, two foliar treatments of biostimulator F-Aspir (5 L ha^–1^) were applied. Finally, in terms of irrigation, drip irrigation was used in Carrizales and Moncada, whereas in La Punta, water was supplied using flood irrigation once every 2 weeks.

### Soil and Water Conductivity

For each assayed field, 10 soil samples from different sites were collected, and their conductivity was measured. The soil samples were homogenized and dried at room temperature. The dry soil samples were then sieved (2 mm) and the soil conductivity (dS m^–1^) was determined using the method described by Primo and Carrasco (1980). The conductivity of the water supply was measured in collected water samples with an electrical conductivity meter (CM35, CRISON, Barcelona, Spain).

### Pathogen Detection

All the plants showing virus symptoms were sampled, and the viruses were identified following Pérez-de-Castro et al. (2019). Root samples were obtained from plants that showed symptoms of soilborne pathogens and they were analyzed to identify the causal agents. Small pieces (0.5–1 cm) from the cortical necrosis of both lower stem and upper root were surface disinfected for 1 min in 1.5% NaClO, washed four times with sterilized bi-distilled water, and plated onto potato dextrose agar (PDA) amended with streptomycin sulfate (0.5 g/L) to avoid bacterial contamination. Plates were incubated at 25°C in the dark for 3–5 days. Then, emerging colonies were transferred to 6 cm Ø PDA plates, and each isolate was subcultured to get pure cultures for subsequent characterization.

Fungal isolates obtained were identified and characterized morphologically on the basis of comparison of their different somatic and/or sexual and asexual reproductive structures. A molecular characterization was made by PCR amplification of the ribosomal ITS fragment for most of the isolates, and TEF-1α and RPB2 gene fragments in the case of certain *Fusarium* species, using ITS1/ITS4 (White et al., 1990), EF1/EF2 (O’Donnell et al., 1998), and fRPB2-7cF/fRPB2-11aR (Reeb et al., 2004) primers, respectively. Sequences obtained allowed the identification of isolates by their comparison with homologous sequences deposited in public databases like GenBank (using BLASTn tool) or Fusarium ID Database^3^, as well as by performing phylogenetic reconstructions employing Bayesian inference methods from multilocus alignments of combined genomic regions for some of the mentioned *Fusarium* taxa.

### Pathogenicity Tests Against Fungal Pathogens

The degree of susceptibility/tolerance of the snake melon cultivar against four of the most frequently isolated soilborne pathogens, the fungi *Neocosmospora keratoplastica* and *Neocosmospora falciformis*, recently detected causing Fusarium wilt in melon in Spain for the first time (González et al., 2020b, c), and *M. cannonballus* and *Macrophomina phaseolina*, previously reported to cause severe vine decline and charcoal root rot in melons (Castro et al., 2020; de Sousa Linhares et al., 2020), was evaluated. Isolates from Carrizales were used for *Neocosmospora* spp. and from La Punta for *M. cannonballus* and *M. phaseolina*, respectively.

For *N. keratoplastica* and *N. falciformis*, plants of the snake melon cultivar and “Don Quixote” Piel de Sapo (commercial control not previously tested against these pathogens) were grown with a sterilized substrate in plastic trays. When plants reached the 15-day-old stage, they were uprooted and artificially inoculated by root dip for 2 min into a conidial suspension of 5 × 10^6^ conidia ml^–1^ of each isolate (seven plants with each pathogen and three non-inoculated controls). Then, inoculated plants were planted into pots containing sterilized substrate and maintained in a growth chamber for 30 days at 26°C (González et al., 2020c). Disease severity was evaluated at 30 days after inoculation (DAI), using the following scale: 1 = no symptoms; 2 = beginning of wilting or yellowing on leaves; 3 = all leaves completely wilted, stem standing; and 4 = dead plant (Cothiere et al., 2016). Plants with a disease severity score (at 30 DAI) lower than 2 were considered to be resistant (R); between 2 and 3, moderately resistant (MR); and higher than 3, susceptible (S). The area under the disease progress curve (AUDPC) was calculated for each inoculation (Perchepied and Pitrat, 2004) with the formula:

$$\text{AUDPC}=\sum_{i}\left[\left(x_{i}+x_{i+1}-2\right)/2\right]\left(t_{i+1}-t_{i}\right)$$

where *i* = 1 to 4 scorings, *x*_*i*_ = mean disease score of each plant at date *i*, *x*_*i* + 1_ = mean disease score of each plant at date *i* + 1, and *t*_*i* + 1_−*t*_*i*_ = number of days between scoring date i and scoring date *i* + 1. The AUDPC value is effective for determining the progress of the disease; it gathers different observations during the epidemic and summarizes all the values in a single one that reflects the severity of disease. Finally, dead or severely wilted plants were removed and processed to check the cause of death and fulfill Koch’s postulates.

For both *M. cannonballus* and *M. phaseolina*, six plants of the snake melon accession were inoculated and three were used as non-inoculated controls. Also, six plants of the “Piñonet” Piel de Sapo cultivar were used as commercial susceptible control (Ambrósio et al., 2015; Castro et al., 2020). The inoculum of *M. cannonballus* was prepared as described in Castro et al. (2020). Briefly, inoculated PDA plates were incubated at 25°C for 1 week and used to inoculate autoclaved bottles with hydrated wheat seeds. These bottles were incubated at 25°C for 1 month, being shaken weekly. After this period, the final inoculum was prepared by mixing 200 g of the inoculated wheat seeds kg^–1^ of peat. Plants were grown in a greenhouse, and 30 DAI roots were washed and extended on an acetate film. Root damage of the primary and lateral roots was scored from 0, healthy with no lesions, to 4, highly damaged roots with root rots, rootlets pruning, etc. The inoculum of *M. phaseolina* was prepared as reported in Ambrósio et al. (2015). Briefly, the isolate was inoculated in PDA + antibiotic (tetracycline 0.05 g/L) plates. This culture was used to inoculate plates containing toothpicks in PDA medium that was incubated at 28°C for 1 week. Inoculated toothpicks were inserted at the base of the stem of plants 20 days after transplanting. Plants were grown in a greenhouse, and the severity of the infection was scored at 7 and 15 DAI, with a severity index, from 0, asymptomatic, to 4, severe lesions on the stem.

### Fruit Characterization

**FIGURE 1:**
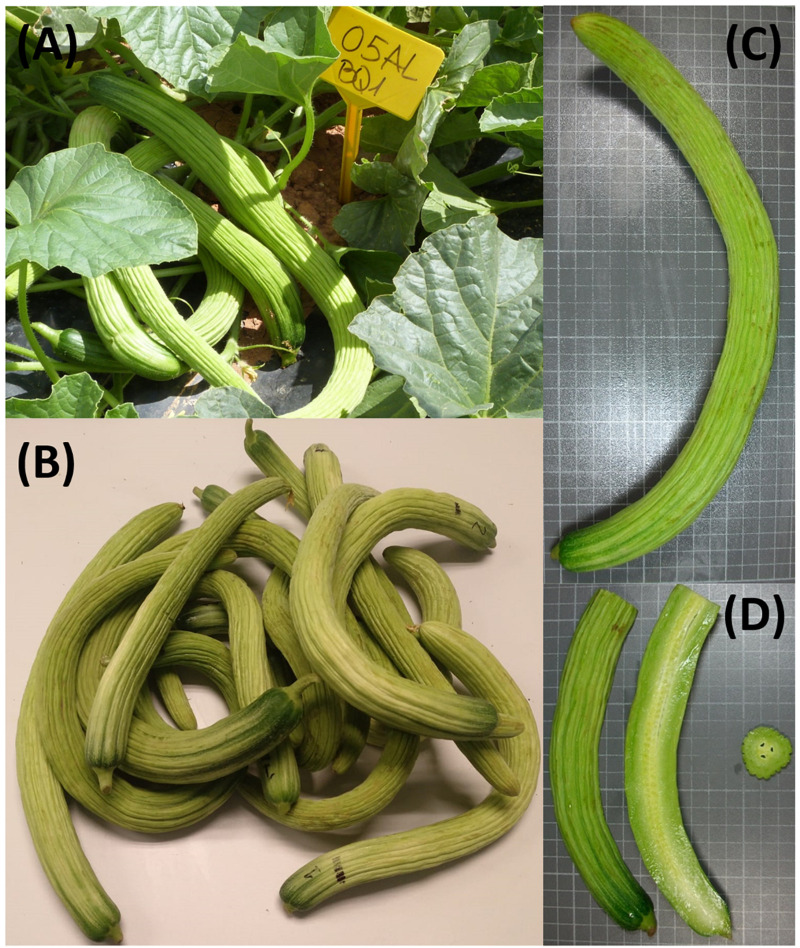
Snake melon fruit in the field ready to be harvested **(A)** and harvested fruit **(B)**. Characterization of snake melons **(C,D)**.

All fruits of marketable size were weighted at the time of harvest to estimate total marketable yield per plant (Figures 1A,B). Two fruits per plant were selected for characterization (Figures 1C,D) in order to obtain data for fruit weight (FW, g, measured with a digital scale), fruit length and diameter (FL and FD, in cm, measured with a graduated ruler), rind and flesh firmness (RF and FF, measured with a penetrometer in kg cm^–2^), fruit pH (universal pH indicator paper), and soluble solids content [SSC, measured as °Brix from drops of juice using a hand-held *Pocket* refractometer (PAL-α), Atago CO., LTD, Tokyo, Japan]. Both fruit flesh and rind colors were measured with a CR-400 colorimeter (Konica Minolta, Inc., Tokyo, Japan), obtaining Hunter L, a, and b coordinates.

### Fruit Sensorial and Metabolomics Analysis

Sensory evaluations were performed employing a modified methodology of Saftner et al. (2006).

In 2018, four sensory evaluations using a consumer panel (20 tasters) were performed with snake melon fruits from La Punta (evaluations 1 and 2) and Carrizales (evaluations 3 and 4). In each evaluation, the fruits harvested from different plants of each treatment were arranged in three biological replicates for each of the three treatments, NG and grafted onto F_1_Pat81 and Cobalt. The panelists were asked to score the nine samples randomly arranged (three biological replicates of each of the three treatments) in a 1–5 scale (5 representing the highest acceptability), considering flavor, texture, and aroma together. During the evaluations, panelists could add extra comments regarding the scores provided. Panelists were given water and low salt cracker to cleanse the palate. All snake melon samples were collected the day of the evaluation, as snake melons have a short shelf life.

In 2019, five sensory evaluations (20 tasters) were performed, the first three using fruits obtained from La Punta and the last two from Carrizales. The same design as that in 2018 was used, and fruits harvested from different plants of each treatment were arranged in three biological replicates. Evaluations 1 and 5 compared fruits from NG plants and plants grafted onto the two *Cucumis* rootstocks, F_1_Pat81 and Fian. Evaluations 2, 3, and 4 compared fruits from NG plants and grafted onto the *Cucumis* and *Cucurbita* rootstocks, F_1_Pat81 and Shintoza. Panelists were asked to provide a score between 1 and 5 to flavor, texture, and aroma to each of the nine samples randomly arranged (three biological replicates of each treatment). Aliquots of fruit tissue from the biological replicates used for the sensory evaluations were used for sugars, organic acids, and volatile organic compounds (VOCs) analysis.

### Sugar and Acid Analysis

The aliquots of fruit tissue from the biological replicates used in sensory evaluations of year 2019 (La Punta and Carrizales, NG plants and plants grafted onto F_1_Pat81, Fian, and Shintoza) were homogenized (Silent Crusher M; Heidolph, Schwabach, Germany) and frozen at −80°C until analysis. A half of each sample was used for sugars (sucrose, glucose, and fructose) and organic acids (citric, malic, and glutamic) analysis. Only sporadic contents of glutamic acid were found, and these data were not included in the results. These compounds were quantified following the methodology described by Cebolla-Cornejo et al. (2012) based in capillary electrophoresis. For that purpose, an Agilent 7100 system (Agilent Technologies, Waldbronn, Germany) was used.

For the analysis, samples were thawed in a refrigerator in complete darkness. Then, they were centrifuged at 510 × *g* for 5 min. The upper phase was diluted (1:20) with deionized water and filtered using centrifuge tube filters with 0.22-μm membranes (Costar^®^ Spin-X^®^, Corning, Amsterdam). For the separation, fused-silica capillaries (Polymicro Technologies, Phoenix, AZ, United States) with 50 μm internal diameter, 363 μm external diameter, 67 cm total length, and 60 cm effective length were used. They were previously conditioned with flushes at 95,000 Pa of NaOH 1 mol L^–1^ at 50°C for 5 min, NaOH 0.1 mol L^–1^ for 5 min at 20°C, and deionized water (Elix 3, Millipore, Billerica, MA, United States) for 10 min. At the beginning of each sequence, the capillary was flushed at 20°C with the running background electrolyte (BGE) for 30 min. BGE consisted of 20 mmol L^–1^ 2,6-piridin dicarboxylic acid at pH 12.1 and 0.1% w:v hexadimethrine bromide. Between runs, the capillary was flushed with 58 mmol L^–1^ SDS (2 min) and BGE (5 min). Samples were injected hydrodynamically at 3400 Pa for 10 s, and separations were performed at −25 kV and 20°C. Absorbance was measured at 214 nm. Results were expressed in g kg^–1^ fresh weight (FW). Sucrose equivalents were calculated by multiplying sucrose, glucose, and fructose contents by their relative sweetening power, 1, 0.74, and 1.73, respectively, and adding them up (Koehler and Kays, 1991).

### Analysis of Volatile Compounds

The second half of each homogenized sample used in sugar and acid analysis was employed for the analysis of VOCs listed in Supplementary Table 1. The method described by Fredes et al. (2017) was followed. Solid phase extraction (SPE) cartridges were conditioned with 5 ml of diethyl ether, 5 ml of n-hexane, and dried for 10 min. Frozen samples were defrosted in the fridge. Once thawed, 30 g of the sample was weighed into a 150-ml Erlenmeyer flask with a stopper. The extraction was carried on using a purge and trap headspace system, using nitrogen gas for the inlet tube and the conditioned SPE cartridge for the outlet tube. The samples were extracted for 49 min at 40°C using magnetic agitation and nitrogen flow of 1.6 ml min^–1^. Subsequently, the cartridges were eluted using 5 ml of a diethyl ether-hexane 1:1 (v:v) solution and 5 ml of diethyl ether. Finally, the collected elution solvents were evaporated to 0.5 ml at 35°C under a nitrogen flow. The resulting extracts were divided into two aliquots in sealed Gas Chromatography (GC) vials, and frosted at −40°C until analysis.

The chromatographic analysis of VOCs was carried on using a Shimadzu GC-2010 Plus system (Shimadzu, Kioto, Japan) coupled with a single quadrupole mass spectrometry system (GCMS QP2010 Ultra, Shimadzu, Kioto, Japan). A Supelcowax 10 column of 30 m × 0.25 mm (Sigma-Aldrich, St. Louis, MO, United States) was used. Helium was used as carrier gas at a flow of 1 ml min^–1^. The injection was performed in split mode (split ratio 1/50) with a volume of injection of 1 μl at 250°C. The temperature program started at 30°C during 4 min after the injection followed by a rise to 160°C (10°C min^–1^), and finally, a rise to 250°C (30°C min^–1^), which was maintained for 3 min. The mass spectra were acquired in Selected ion monitoring (SIM) mode using the m/z for each compound. Electron ionization in positive mode was used at a temperature of 250 and 230°C for the interphase and the ion source, respectively.

From those treatments included in the five sensory evaluations of 2019, NG and F_1_Pat81, 10 biological replicates were analyzed for sugar and VOC contents (two of the three biological replicates used per each sensory evaluation), and 7 and 4 biological replicates were independently analyzed for the Shintoza and Fian treatments that were included in three and two of the sensory evaluations performed, respectively.

### Statistical Analysis

Fruit characterization, agronomic, sugars, acids, and volatiles data were analyzed using a Dunnett’s test. For each location, the effect of each rootstock was compared to the NG control. StatGraphics Centurion version 17.2.04 for Windows and IBM SPSS Statistics 25 for Windows were used for this purpose. Principal component analysis (PCA) of VOCs data were performed using S-Plus v. 8.01 for Windows (Insightful Corp., Seattle, WA, United States). A biplot representation was then obtained, including the scores of data points and the loadings of each VOC for each principal component. Pairwise correlations for metabolite and sensory analysis data were graphically represented as heatmaps using the software *heatmapper*^4^.

## Results

### Growth-Limiting Factors

#### Climate, Water, and Soil Properties

Mean temperatures were similar in the three fields (Supplementary Figure 3) except a significantly higher temperature (around 1°C) in April in Carrizales and La Punta compared to Moncada in 2018, and higher temperature in June and July 2019 in Carrizales compared to La Punta. Rainfall was higher in La Punta in the middle of the growing cycle (June) in 2018 and at the beginning (April) of the 2019 assay, and in Carrizales at the end of the growing cycle in 2019.

The irrigation water of Carrizales showed high values of conductivity, reaching 4.5 dS m^–1^ in 2018 and 5.95 dS m^–1^ in 2019. Thus, the high salinity in this area of cultivation was confirmed. In fact, soil conductivity reached values of 3.17 ± 0.05 dS m^–1^ in 2018 and 1.66 ± 0.10 dS m^–1^ in 2019. The other two fields showed rather standard values for the area, with water conductivity below 2.2 dS m^–1^ and soil conductivity under 0.7 dS m^–1^ in both years.

#### Pests and Diseases

Aphids were the main pest in all the fields in the 2 years, with a higher incidence in La Punta and Moncada than in Carrizales (Supplementary Figure 4).

During 2018, in La Punta, some snake melon plants showed mosaic symptoms (Supplementary Figure 4) and the aphid borne potyvirus WMV was detected, with 9% of snake melons affected by this virus. This year in Moncada, the presence of *Cucumber mosaic virus* (CMV), also transmitted by aphids, was not detected in snake melons, although it was present in sweet melons cultivated in the same field. No viruses were detected in the plants grown in Carrizales, likely associated to a reduced presence of the insect vectors. *Podosphaera xanthii*, the fungus responsible for the cucurbit powdery mildew, was detected in La Punta and Moncada, but only caused mild infections to the snake melon plants, whereas the sweet melons were severely affected.

In 2019, WMV was also the most prevalent virus in La Punta, with 26% of the snake melon plants affected by the virus. Other aphid-transmitted potyviruses such as ZYMV and the whitefly transmitted Begomovirus ToLCNDV, both affecting approximately 18% of the snake melon plants, were also detected in this field. *P. xanthii* (Supplementary Figure 4) affected the snake melon plants in La Punta. Again, the field of Carrizales was free of virus and powdery mildew.

In accordance with the history of fungal stress, we observed some mortality of snake melon plants with vine decay symptoms likely due to fungal soilborne pathogens in 2018 in La Punta. Twelve percent of the NG plants of snake melon died at early developmental stages, and *M. phaseolina* was isolated from their roots. Twelve percent of the snake melon plants grafted onto the *Cucurbita* rootstock Cobalt died at later stages, with roots affected by *Fusarium oxysporum*, whereas plants grafted on F_1_Pat81 were less affected by fungi. Other soilborne pathogens, detected in roots of sweet melon plants cultivated in the same field, but not affecting snake melons, were *Fusarium equiseti*, *N. falciformis*, *Fusarium solani*, *F. solani* f. sp. *cucurbitae*, *M. cannonballus*, *Torula herbarum*, *Fusarium incarnatum*, *Rhizopus* sp., *Alternaria alternata*, *Alternaria* sp., *Rhizoctonia solani*, *Plectosphaerella cucumerina*, *Aspergillus flavus*, *Aspergillus* sp., *Fusarium chlamydosporum*, *Acremonium* sp., *Geotrichum candidum*, *Gibberella fujikuroi*, *Gibberella* sp., *Chaetomium acropullum*, *Chalaropsis radicicola*, *Collariella bostrychodes*, and *Penicillium* sp.

In 2018, no snake melon plants were affected by soilborne diseases in Carrizales, although different soilborne pathogens were detected in the roots of sweet melon plants cultivated in the same field (*M. phaseolina*, *N. falciformis*, *N. keratoplastica*, *F. solani*, *M. cannonballus*, *F. oxysporum*, *Alternaria* sp., *Cladosporium herbarum*, *Fusarium* sp., *C. bostrychodes*, *Mortierella alpina*, and *Acremonium* sp.). No fungal stress was observed in Moncada.

The fungal attack in 2019 was less severe in La Punta, where the lower mean temperature in May, June, and August and higher rainfall by the end of the cycle could have contributed to the reduced fungal damage (Supplementary Figure 3). No snake melon plant died by soilborne pathogens. Although some soilborne pathogens were detected in the sweet melon plants cultivated on the field (*M. phaseolina*, *F. oxysporum*, *F. equiseti*, *F. solani*, *Fusarium* sp., *N. falciformis*, *Alternaria tenuissima*, *A. alternata*, *Gibberella* sp., *Pyxidiophora arvernensis*, *Botryosphaeriaceae*, *C. herbarum*, *Pythium aphanidermatum*, *Gibberella avenacea*, and *Mucor* sp.), they did not affect the snake melon plants.

This year, a more severe fungal attack was observed in Carrizales (Supplementary Figure 5) that might be associated to the different characteristics of the cultivation plot and to the higher average temperatures in July along with a reduced rainfall until the end of the growing cycle compared to the previous year (Supplementary Figure 3). The snake melons showing symptoms of soilborne pathogens were mainly those grafted onto *Cucurbita* rootstocks, Shintoza and Cobalt, and the NG plants (44, 31, and 25% mortality, respectively). The main pathogens detected were *M. phaseolina*, *N. falciformis*, *F. equiseti*, *Gibberella* spp., and *Fusarium longipes*. No symptoms of fungal attack were observed in plants grafted onto *Cucumis* rootstocks, F_1_Pat81, Fimy, and Fian. Sweet melons planted on Carrizales were also affected by the same pathogens.

### Response of Snake Melon to *M. phaseolina*, *M. cannonballus*, and *Neocosmospora* spp.

**FIGURE 2:**
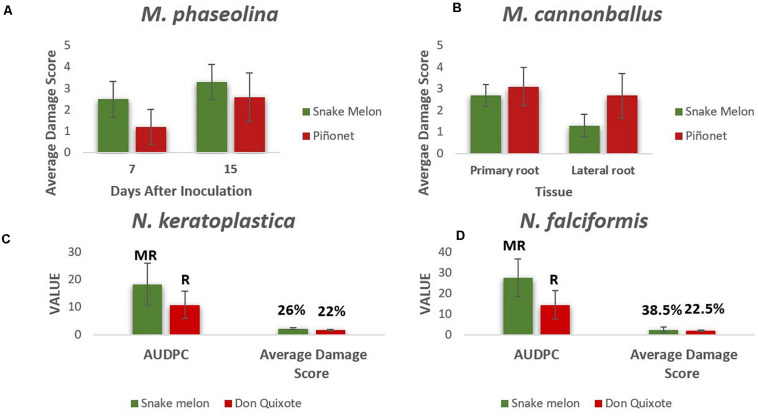
**(A)** Average damage scores (from 0 to 4) of the stem lesions caused by *M. phaseolina* in snake melon and sweet melon control (Piel de Sapo Piñonet) at 7 and 15 days after inoculation (DAI). **(B)** Average damage scores (from 0 to 4) caused by *M. cannonballus* in primary and lateral roots of snake melon and sweet melon control at 30 DAI. Average area under the disease progress curve (AUDPC), average damage score, type of reaction (R, resistant; MR, moderately resistant), and disease incidence (%) for snake melon and the sweet melon control plants (Piel de Sapo Don Quixote) inoculated with *N. falciformis* **(C)** and *N. keratoplastica* **(D)**. Six **(A,B)** and seven **(C,D)** biological replicates (plants) were used for each genotype.

The field survey results indicated that the main pathogen associated with dead snake melon plants or plants showing decay symptoms in our conditions is *M. phaseolina*. To test the level of susceptibility to this pathogen under controlled conditions, we conducted an artificial inoculation assay, using a *M. phaseolina* isolate collected in La Punta. Results showed that snake melon is highly susceptible to this pathogen. Symptoms started at 7 DAI and were severe at 15 DAI (Figure 2A). Average damage score was higher in snake melons than in the control sweet melon Piel de Sapo that has also proven to be susceptible to this disease (de Sousa Linhares et al., 2020). All the snake melon plants were dead after 30 DAI, while the mortality of the susceptible control Piel de Sapo was 20%.

We also tested the response to *M. cannonballus*, despite the fact that it was less frequent than *Macrophomina* in our fields, because in many hot and dry regions of melon cultivation, these are two of the main soilborne pathogens affecting melons (Cohen et al., 2016). Snake melon plants were also susceptible to *M. cannonballus* (Figure 2B), with similar average damage scores in primary roots, although less severe lesions in the lateral roots compared to Piel de Sapo.

Field results also showed the importance of *Fusarium* species. Snake melons are known to be highly susceptible to several pathogenic forms of the so-called *F. oxysporum* species complex (Solmaz et al., 2016b; Al-Taae and Al-Taae, 2019), but their response to other species belonging to the *F. solani* species complex (FSSC) (e.g., some of them currently included in the genus *Neocosmospora*) had not been tested yet. For both pathogens, *N. falciformis* and *N. keratoplastica*, snake melon plants showed considerably higher AUDPC than the commercial control sweet melon Piel de Sapo Don Quixote, as well as a higher disease incidence for *N. falciformis* (Figures 2C,D). Supplementary Figures 6, 7 show symptoms on snake melon caused by the different pathogens.

### Yield and Fruit Characteristics

**FIGURE 3:**
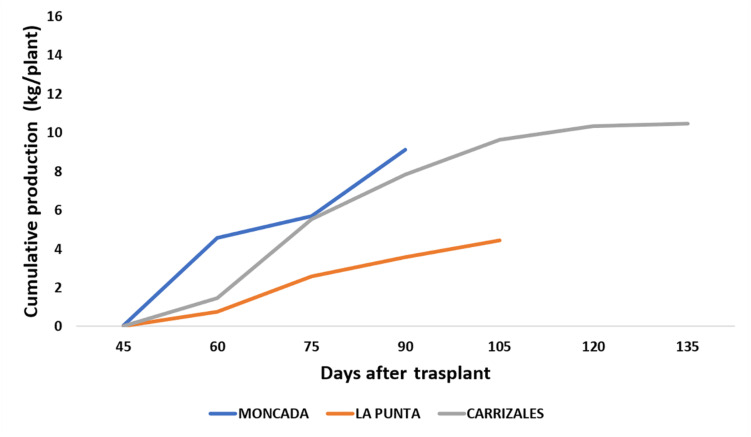
Cumulative production (kg plant^–1^) of non-grafted (NG) snake melons in 2018, cultivated in the three fields. Sixteen NG plants were cultivated per field. All fruits of marketable size were weighted at the time of harvest to estimate total yield per plant.

The agronomic performance of NG melons in the three fields was compared to analyze the effect of the agro-ecological conditions (Figure 3 and Table 1). Moncada, representing unstressed conditions, could be considered as control, as the lack of previous melon cultivation led to the absence of soilborne diseases, while La Punta and Carrizales represented stressful conditions due to the incidence of diseases and the use of saline water and soil, respectively. The yield of NG snake melon plants in La Punta was reduced compared to Moncada (≈ 4 kg/plant vs ≈ 10 kg/plant, respectively) (Figure 3). This reduction was likely a consequence of the incidence of viruses and soilborne diseases, as stated above. Conversely, the high salinity conditions at Carrizales did not affect yield per plant, which was similar to that of Moncada. No differences were found for most fruit traits among the three fields (Table 1), although the stressful conditions of both La Punta and Carrizales resulted in lower fruit FF. Also, regarding basic quality characteristics, the SSC was higher at Carrizales, which is expected under saline conditions. At this field, fruits showed higher Hunter a value, representing a less greenish color of the flesh.

**TABLE 1:** Agronomic performance and fruit characterization of non-grafted (NG) plants in the three fields (Moncada, La Punta, and Carrizales) in 2018.

	Field		
	Moncada	La Punta	Carrizales
Plant mortality (%)	0	12	0
Fruit weight (g)	243.2 ± 18.2	276.4 ± 25.5	251.1 ± 37.6
Fruit length (cm)	48.9 ± 2.0	43.7 ± 2.0	42.7 ± 1.7
Fruit diameter (cm)	3.0 ± 0.1	3.3 ± 0.2	3.0 ± 0.1
Flesh firmness (kg cm^–2^)	4.2 ± 0.3	2.6 ± 0.4*	3.1 ± 0.2*
Rind firmness (kg cm^–2^)	10.0 ± 0.6	9.8 ± 1.1	8.4 ± 0.5
pH	5 ± 0	5 ± 0	5 ± 0
Soluble solids content (°Brix)	3.3 ± 0.2	3.6 ± 0.2	4.0 ± 0.2*
Hunter L (F)	60.22 ± 1.34	55.56 ± 1.35	65.81 ± 1.79
Hunter a (F)	−11.64 ± 0.27	−11.43 ± 0.39	−10.2 ± 0.27*
Hunter b (F)	20.56 ± 0.52	19.81 ± 0.73	20.89 ± 1.48
Hunter L (R)	48.02 ± 1.4	51.98 ± 1.56	52.69 ± 1.56
Hunter a (R)	−13.04 ± 0.37	−12.13 ± 0.53	−12.97 ± 0.62
Hunter b (R)	20.49 ± 0.51	19.45 ± 0.82	20.29 ± 0.72

Hunter’s L, a, b color coordinates measured in the flesh (F) and rind (R). Values followed by a (*) are significantly different from that of the unstressed field (Moncada) using Dunnett’s test (P ≤ 0.05). Two fruits per plant (from those plants of the 16 cultivated that remained alive at the time of harvest) were characterized.

**FIGURE 4:**
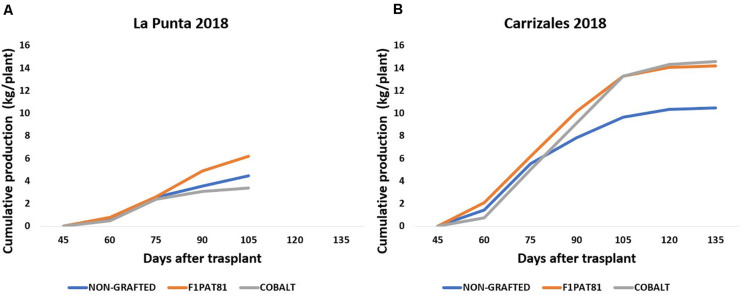
Cumulative production (kg plant^–1^) of snake melons in 2018 grown in La Punta **(A)** and Carrizales **(B)** obtained from non-grafted plants and plants grafted onto the commercial Cucurbita hybrid Cobalt and the experimental *C. melo* hybrid F_1_Pat81. Sixteen plants were cultivated per field and treatment. All fruits of marketable size were weighted at the time of harvest to estimate total yield per plant.

The effect of grafting during 2018 was evaluated in the fields of La Punta and Carrizales using the commercial *Cucurbita* hybrid Cobalt and the experimental *C. melo* hybrid F_1_Pat81. Some mortality was observed in grafted snake melon plants in La Punta, with the F_1_Pat81 rootstock being the one that displayed the best performance under these epidemiological conditions. The plants grafted onto this rootstock displayed less mortality and higher yield ≈ 6 kg/plant compared to the ≈ 4 kg/plant (Figure 4A) produced by the plants grafted onto Cobalt and NG. The higher susceptibility of snake melon roots and the Cobalt rootstock to the fungal pathogens may account for these differences. Production in Carrizales (Figure 4B) was higher than in La Punta. In this field, fungal stress was lower, and the salt stress had a much less severe impact on snake melon production. Grafting had a favorable effect on snake melon production under saline conditions. The plants grafted onto the melon rootstocks F_1_Pat81 were as productive as those grafted onto Cobalt (≈ 14 kg/plant), both much more productive than NG plants (≈ 10 kg/plant).

Regarding snake melon fruit traits, no differences were found between fruits from the NG and those of grafted for fruit shape, firmness, pH, and °Brix (Table 2). Only Hunter L and a values were altered in fruits from plants grafted on F_1_Pat81 in La Punta. They were less green with a lighter color. Lower Hunter a values were obtained in fruits from plants grafted onto Cobalt in Carrizales (more greenish color). As stated before, the saline conditions of Carrizales produced snake melon fruits with higher °Brix in all treatments (Table 2).

**TABLE 2:** Agronomic performance and fruit characterization of the snake melons produced in plants grafted onto commercial *Cucurbita* hybrid Cobalt and experimental *C. melo* hybrid F_1_Pat81 compared to non-grafted in two fields (La Punta and Carrizales) in 2018.

		Treatment		
	Field	NG	Cobalt	F_1_Pat81
Plant mortality (%)	Carrizales	0	0	0
	La Punta	12	12	0
Fruit weight (g)	Carrizales	251.1 ± 37.6	204.9 ± 26.3	200.3 ± 20.8
	La Punta	276.4 ± 25.5	242.8 ± 29.1	259.9 ± 44.5
Fruit length (cm)	Carrizales	40.7 ± 1.7	38.4 ± 0.8	36.4 ± 0.8
	La Punta	43.7 ± 2.0	38.8 ± 0.7	40.1 ± 0.7
Fruit diameter (cm)	Carrizales	3.0 ± 0.1	2.9 ± 0.2	2.7 ± 0.1
	La Punta	3.3 ± 0.2	3.1 ± 0.1	3.2 ± 0.2
Flesh firmness (kg cm^–2^)	Carrizales	3.1 ± 0.2	3.7 ± 0.7	3.2 ± 0.4
	La Punta	2.6 ± 0.4	2.7 ± 0.4	2.4 ± 0.2
Rind firmness (kg cm^–2^)	Carrizales	8.4 ± 0.5	9.8 ± 0.9	8.3 ± 0.9
	La Punta	9.8 ± 1.1	9.8 ± 1.0	8.8 ± 0.5
pH	Carrizales	5 ± 0	5 ± 0	5 ± 0
	La Punta	5 ± 0	4.88 ± 0.13	4.75 ± 0.16
Soluble solids content (°Brix)	Carrizales	4.1 ± 0.2 -/*	4.0 ± 0.2 -/*	3.8 ± 0.1 -/*
	La Punta	3.6 ± 0.2 -/*	3.5 ± 0.2_/*	3.2 ± 0.2 -/*
Hunter L (F)	Carrizales	65.81 ± 1.79 -/*	58.85 ± 2.1	70.11 ± 2.58 -/*
	La Punta	55.56 ± 1.35 -/*	56.86 ± 1.83	62.03 ± 1.09 */*
Hunter a (F)	Carrizales	−10.22 ± 0.26	−11.66 ± 0.32 */-	−10.38 ± 0.24
	La Punta	−11.43 ± 0.38	−11.70 ± 0.4	−9.75 ± 0.64 */-
Hunter b (F)	Carrizales	20.89 ± 1.48	20.29 ± 0.43	21.62 ± 1.77
	La Punta	18.81 ± 0.73	20.07 ± 0.51	18.76 ± 0.73
Hunter L (R)	Carrizales	52.69 ± 1.56	48.6 ± 0.64	49.87 ± 1.88
	La Punta	51.98 ± 1.56	50.57 ± 1.03	52.1 ± 1.05
Hunter a (R)	Carrizales	−12.97 ± 0.62	−13.39 ± 0.17 -/*	−13.68 ± 0.44 -/*
	La Punta	−12.13 ± 0.53	−12.16 ± 0.34 -/*	−11.07 ± 0.24 -/*
Hunter b (R)	Carrizales	20.29 ± 0.71	20.06 ± 0.38	19.72 ± 0.58
	La Punta	19.45 ± 0.82	19.8 ± 0.5	18.96 ± 0.35

Hunter’s L, a, b color coordinates measured in the flesh (F) and rind (R). Values followed by a (*/-) show significant differences compared to the non-grafted control (Dunnett’s test; P ≤ 0.05). Values followed by a (-/*) show significant differences compared between the two fields for the same treatment (P ≤ 0.05). Values followed by (*/*) indicate a significant difference compared to the non-grafted control and between the two fields (Dunnett’s test; P ≤ 0.05). Two fruits per plant (from those plants of the 16 cultivated that remained alive at the time of harvest) were characterized.

**FIGURE 5:**
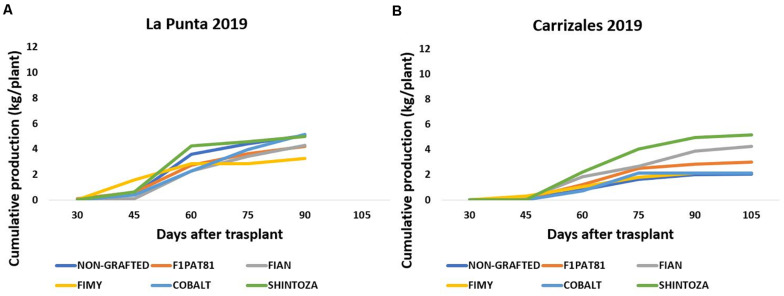
Cumulative production (kg plant^–1^) of snake melons in 2019 from non-grafted plants and plants grafted on commercial *Cucurbita* hybrids (Cobalt and Shintoza), the *C. melo* hybrid F_1_Pat81, and hybrids between *C. ficifolius* and *C. anguria* (Fian) or *C. myriocarpus* (Fimy) in the two sites of cultivation La Punta **(A)** and Carrizales **(B)**. Sixteen plants were cultivated per field and treatment. All fruits of marketable size were weighted at the time of harvest to estimate total yield per plant.

In 2019, the fungal stress in La Punta did not cause plant mortality in snake melons. However, despite lower mortality, the impact on yield was important (Figure 5A). Yield per plant ranged from ≈ 4 to 5 kg plant^–1^, except for the snake melon plants grafted onto the Fimy rootstock that was the less productive. In Carrizales, the yield in 2019 dropped significantly compared to 2018 (Figure 5B). The fungal attack was more severe. Also, this year, conductivity of irrigation water was higher. Additionally, the fact that an extensive crop like oat, and not a nitrogen fixing crop like alfalfa, was cultivated for the three previous years might have resulted in a poorer and less productive soil. The fungal attack resulted in a higher plant mortality in both NG and grafted onto *Cucurbita* rootstocks plants, whereas *Cucumis* rootstocks were again the most tolerant to fungi. Among them, the wild Fian and the cultivated hybrid F_1_Pat81 maintained a moderate yield per plant compared to NG plants. Fimy was again the less productive rootstock.

No significant effects were found regarding fruit traits in grafted plants compared to the NG ones, except for a reduction in °Brix of the fruits harvested from plants grafted onto Shintoza in Carrizales (Table 3). Again, differences between fields were mainly associated with °Brix, higher in Carrizales, and fruit color, with a more yellowish fruit with less firm flesh in La Punta (Table 3).

**TABLE 3:** Agronomic performance and fruit characterization of the non-grafted control compared to plants grafted onto two commercial *Cucurbita* hybrids (Cobalt and Shintoza), the *C. melo* hybrid F_1_Pat81, and hybrids between *C. ficifolius* and *C. anguria* (Fian) or *C. myriocarpus* (Fimy) grown in the two sites of cultivation (La Punta and Carrizales) in 2019.

		Treatment					
		NG	Cobalt	Shintoza	F_1_Pat81	Fian	Fimy
Plant mortality (%)	Carrizales	25	31	44	0	0	0
	La Punta	0	0	0	0	0	0
Fruit weight (g)	Carrizales	241.4 ± 27.0	289.8 ± 46.2	232.6 ± 22.6	264.1 ± 14.1	223.4 ± 27.4	247.2 ± 25.5
	La Punta	260.4 ± 15.8	278.6 ± 25.6	263.4 ± 20.9	300.7 ± 25.2	234.8 ± 21.0	253.0 ± 17.6
Fruit length (cm)	Carrizales	43.5 ± 1.6	47.0 ± 3.5	39.8 ± 2.0	45.7 ± 2.6	39.7 ± 2.1	39.6 ± 1.9
	La Punta	45.6 ± 2.0	44.7 ± 1.8	44.7 ± 2.3	48.7 ± 2.8	42.4 ± 2.5	43.3 ± 1.7
Fruit diameter (cm)	Carrizales	3.2 ± 0.1	3.3 ± 0.2	3.1 ± 0.2	3.4 ± 0.1	3.2 ± 0.1	3.3 ± 0.1
	La Punta	3.3 ± 0.1	3.2 ± 0.2	3 ± 0.1	3.3 ± 0.1	2.9 ± 0.1	3.0 ± 0.1
Flesh firmness (kg cm^–2^)	Carrizales	4.0 ± 0.3 -/*	3.47 ± 0.22	4.32 ± 0.1 -/*	4.02 ± 0.15 -/*	3.83 ± 0.19	4.18 ± 0.22 -/*
	La Punta	3.6 ± 0.1 -/*	3.6 ± 0.1	3.7 ± 0.2 -/*	3.7 ± 0.2 -/*	3.58 ± 0.2	3.4 ± 0.3 -/*
Rind firmness (kg cm^–2^)	Carrizales	10.1 ± 0.2	9.6 ± 0.4 -/*	10.5 ± 0.2	10.7 ± 0.4	10.5 ± 0.3	10.8 ± 0.4
	La Punta	9.4 ± 0.3	11.1 ± 0.4 -/*	9.7 ± 0.2	10.0 ± 0.3	10.7 ± 0.3	9.8 ± 0.5
pH	Carrizales	4.11 ± 0.11	4.17 ± 0.17	4.44 ± 0.18	4.56 ± 0.18	4.33 ± 0.17	4.22 ± 0.15
	La Punta	4.89 ± 0.11	4.78 ± 0.15	4.78 ± 0.15	4.11 ± 0.11	4.67 ± 0.17	4.57 ± 0.2
Soluble solids content (°Brix)	Carrizales	4.8 ± 0.2 -/*	4.0 ± 0.2	3.8 ± 0.2 */-	4.4 ± 0.2 -/*	4.5 ± 0.3 -/*	4.4 ± 0.3 -/*
	La Punta	3.9 ± 0.2 -/*	4.2 ± 0.1	3.8 ± 0.2	3.3 ± 0.3 -/*	3.8 ± 0.2 -/*	4.0 ± 0.2 -/*
Hunter L (F)	Carrizales	57.58 ± 0.8	59.65 ± 1.3	59.78 ± 1.19 -/*	58.36 ± 1.19 -/*	56.12 ± 1.53	57.76 ± 0.7
	La Punta	54.35 ± 1.67	58.97 ± 1.09	55.18 ± 1.44 -/*	55.18 ± 1.4 -/*	59.88 ± 1.23	57.64 ± 1.95
Hunter a (F)	Carrizales	−11.92 ± 0.4	−11.77 ± 0.45	−11.83 ± 0.42	−12.15 ± 0.23	−11.55 ± 0.39	−11.81 ± 0.38
	La Punta	−12.11 ± 0.35	−11.87 ± 0.38	−12.41 ± 0.25	−11.2 ± 0.43	−12.17 ± 0.28	−11.88 ± 0.24
Hunter b (F)	Carrizales	21 ± 0.22	20.05 ± 0.54	20.96 ± 0.38	21.07 ± 0.31	19.78 ± 0.4 -/*	20.82 ± 0.47
	La Punta	20.44 ± 0.5	21.56 ± 0.51	21.04 ± 0.3	19.79 ± 0.61	21.71 ± 0.42 -/*	21.95 ± 0.85
Hunter L (R)	Carrizales	51.12 ± 0.6	49.81 ± 1.48	50.44 ± 0.51	52.13 ± 0.6	52.02 ± 1.13	50.04 ± 1.42
	La Punta	53.75 ± 0.8	53.02 ± 0.46	51.23 ± 0.95	53.67 ± 1.11	52.72 ± 0.79	50.88 ± 0.93
Hunter a (R)	Carrizales	−12.58 ± 0.46	−12.88 ± 0.26	−13.36 ± 0.21	−13.36 ± 0.4	−13.73 ± 0.41	−12.19 ± 0.41 -/*
	La Punta	−13.08 ± 0.3	−13.18 ± 0.25	−13.74 ± 0.23	−12.91 ± 0.29	−12.82 ± 0.31	−13.56 ± 0.2 -/*
Hunter b (R)	Carrizales	19.33 ± 0.64 -/*	19.67 ± 0.27 -/*	20.48 ± 0.27	20.28 ± 0.49	20.81 ± 0.53	19.27 ± 0.74 -/*
	La Punta	21.34 ± 0.42 -/*	21.51 ± 0.35 -/*	21.67 ± 0.37	21.03 ± 0.5	21.19 ± 0.36	20.77 ± 0.56 -/*

Hunter’s L, a, b color coordinates measured in the flesh (F) and rind (R). Values followed by a (*/-) show significant differences compared to the non-grafted control (Dunnett’s test; P ≤ 0.05). Values followed by a (-/*) show significant differences compared between the two sites for the same treatment (P ≤ 0.05). Two fruits per plant (from those plants of the 16 cultivated that remained alive at the time of harvest) were characterized.

### Sensory Evaluation

**FIGURE 6:**
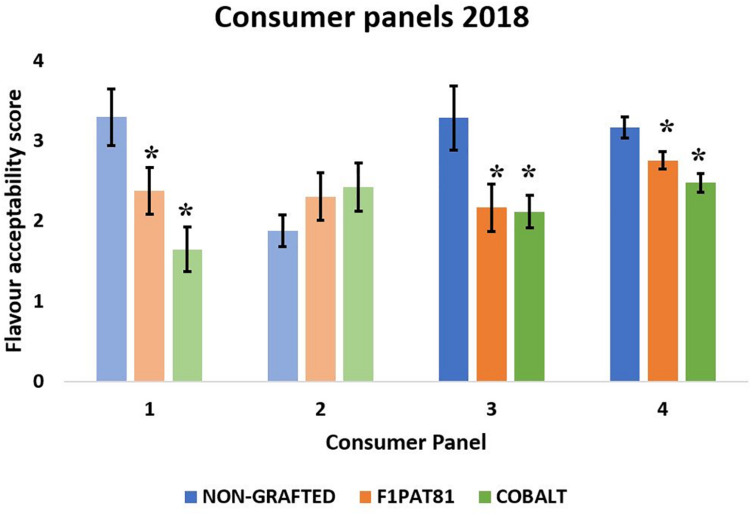
Mean flavor acceptability (1 being the lowest and 5 being the highest) obtained in the sensory evaluations of 2018. Columns of lighter color indicate that the fruit samples were from La Punta and those of darker color indicate that they are from Carrizales. Values followed by (*) show significant differences compared to the NG control (Dunnett’s test, *P* ≤ 0.05). Averages are calculated with the mean scores of 20 panelists on three biological replicates (fruit samples) of each treatment.

Four sensory evaluations were performed in 2018, two with fruits from La Punta and two with fruits from Carrizales (Figure 6). Except for the second evaluation using fruits from La Punta, which did not result in significant differences among treatments, panelists gave higher scores of flavor acceptability to the fruits from NG plants compared to the fruits from plants grafted onto F_1_Pat81 and Cobalt, which had similar scores. Therefore, an effect of grafting on consumer acceptability was found in fruits from both fields.

**FIGURE 7:**
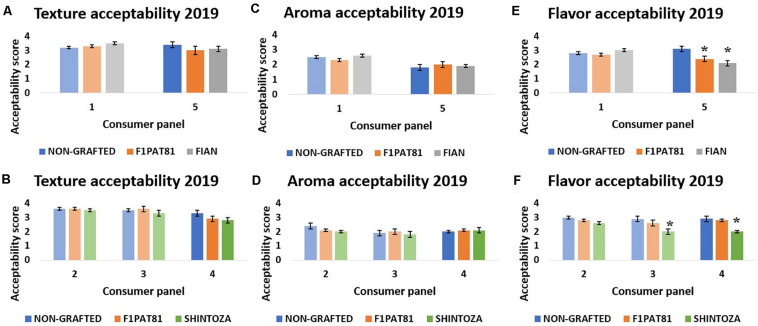
Mean texture, aroma, and flavor acceptability scores (1 being the lowest and 5 being the highest) obtained in the sensory evaluations of 2019. Columns of lighter color indicate that the fruit samples were obtained from La Punta and those of darker color indicate that they were obtained from Carrizales. Columns with (*) show significant differences compared to the NG control (Dunnett’s test, *P* ≤ 0.05). Averages are calculated with the mean scores of 20 panelists on three biological replicates (fruit samples) of each treatment. **(A,C,E)** Correspond to sensory evaluations 1 and 5. **(B,D,F)** Correspond to sensory evaluations 2, 3, and 4.

Five sensory evaluations were performed in 2019 (represented as 1–5 in Figure 7), two comparing fruit from the NG control with those from plants grafted onto two *Cucumis* rootstocks, the melon F_1_Pat81 and Fian (sensory evaluations 1 and 5 for La Punta and Carrizales, respectively), and three comparing the NG with the melon F_1_Pat81 and the *Cucurbita* rootstock Shintoza (sensory evaluations 2 and 3 for La Punta and 4 for Carrizales). This time, flavor, texture, and aroma were scored independently. Significant differences were found for flavor scores, but not for aroma and texture; the Shintoza rootstocks had consistently lower scores compared to NG and F_1_Pat81, and the F_1_Pat81 and Fian fruits from Carrizales were less valued than NG snake melons.

### Accumulation of Sugars, Acids, and Volatiles

**FIGURE 8:**
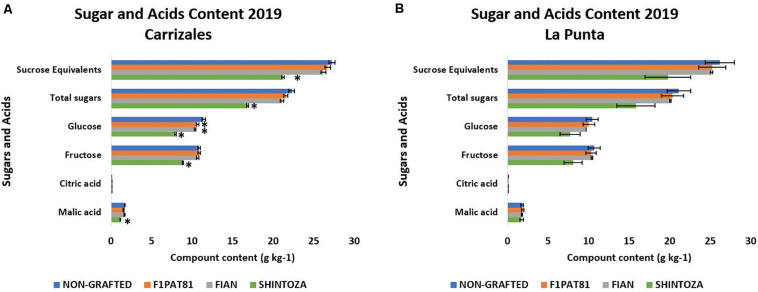
Accumulation of sugars and acids in fruits from non-grafted and grafted (Shintoza, F1Pat81, and Fian) snake melon plants from Carrizales **(A)** and La Punta **(B)**. Columns with (^∗^) show significant differences with respect to the NG control (Dunnett’s test, *P* ≤ 0.05). Averages were calculated from the biological replicates used in the sensory evaluations. From those treatments included in the five sensory evaluations of 2019, NG and F_1_Pat81, averages were calculated from six and four biological replicates, respectively, from the three sensory evaluations of la Punta and the two sensory evaluations of Carrizales. Four and three biological replicates were averaged, respectively, for the Shintoza treatment, included in two sensory evaluations from La Punta and one sensory evaluation from Carrizales. Two biological replicates were averaged for the Fian treatment from each of the two sensory evaluations in which this treatment was included (one from La Punta and one from Carrizales).

Since the sensory evaluations suggested differences in acceptability, we used samples of the evaluation of 2019 to determine sugar and acid accumulation and volatile compounds. The sugar and acid profiles are shown in Figure 8. In general, snake melon fruits have similar levels of fructose and glucose, whereas sucrose levels remained under the limit of detection, expected for a non-sweet fruit. Malic acid was predominant in the samples, with contents up to 20 times higher than those of citric acid.

Grafting had a higher impact in the sugar content than in the organic acid profile (Figure 8). This effect was significant in Carrizales. Although the same trend seemed evident in La Punta, a higher variability hindered the identification of significance. The rootstock that had a higher impact on sugar accumulation was the *Cucurbita* rootstock Shintoza, with a significantly lower amount of both glucose and fructose; malic acid was also reduced with this rootstock. Sugar reduction also occurred, although to a lesser extent, in *Cucumis* rootstocks, both F_1_Pat81 and Fian, but only glucose content was significantly affected. In fact, this reduction did not affect the sucrose equivalents, a variable that accounts for the sweetening power of each sugar and which is more related to sweetness perception.

**FIGURE 9:**
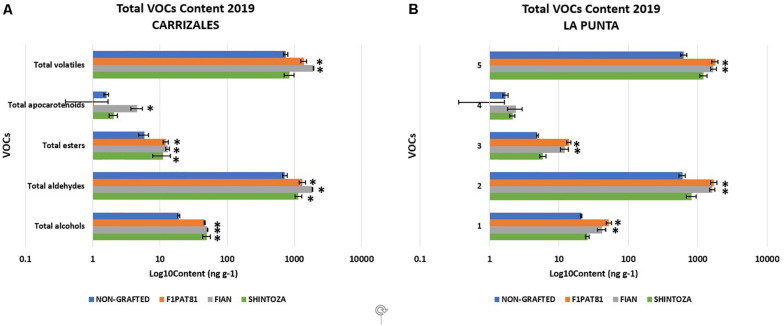
Accumulation of volatile organic compounds in fruits from non-grafted and grafted (Shintoza, F_1_Pat81, and Fian) snake melon plants from Carrizales **(A)** and La Punta **(B)**. Samples obtained from the sensory analyses performed in 2019. Columns with (^∗^) show significant differences with respect to the NG control (Dunnett’s test, *P* ≤ 0.05). Averages were calculated from the biological replicates used in the sensory evaluations as described in legend of Figure 8.

Regarding VOCs, aldehydes represented, by far, the main compounds of the snake melon aroma profile, followed by alcohols, and a very low amount of esters, as expected for a low-aroma melon, and apocarotenoids (Figure 9). The alcohol profile was rich in 2-phenylethanol, associated to floral odor (Table 4). The ester compounds, which are the major contributors to the aroma of sweet melons, were nearly absent in this immature fruit, with ethyl butanoate, the major ester contributor to melon aroma, being the most abundant, although in very low amounts. The apocarotenoid beta-ionone was the only one consistently detected, but in very low amounts.

**TABLE 4:** Detailed accumulation of volatile organic compounds (VOCs) in alficoz fruits from plants non-grafted (NG) and grafted onto F_1_PTA81, Shintoza, and Fian harvested in the 2019 campaign.

			Treatment			
			NG*	Shintoza	F_1_Pat81	Fian
Alcohols (ng g^–1^)	1-Pentanol	Carrizales	n.d.	2.46 ± 0.54 */*	0.94 ± 0.54 -/*	n.d.
		La Punta	n.d.	n.d. -/*	2.26 ± 0.25 */*	0.98 ± 0.98
	(Z)-3-Hexen-1-ol	Carrizales	0.14 ± 0.14 -/*	4.03 ± 0.36 */*	2.45 ± 0.4 */-	2.28 ± 0.04 */-
		La Punta	0.82 ± 0.08 -/*	1.62 ± 0.21 */*	2.89 ± 0.46 */-	1.69 ± 0.41 */-
	1-Nonanol	Carrizales	1.76 ± 0.08 -/*	2.99 ± 0.4 */*	2.61 ± 0.14 */-	3.23 ± 0.09 */-
		La Punta	1.53 ± 0.05 -/*	2 ± 0.17 -/*	2.72 ± 0.16 */-	3.22 ± 0.11 */-
	(Z)-3-Nonen-1-ol	Carrizales	n.d.	n.d.	n.d.	n.d.
		La Punta	0.08 ± 0.08	n.d.	n.d.	n.d.
	Benzyl alcohol	Carrizales	0.29 ± 0.17	3.06 ± 0.71 */*	2.94 ± 0.53 */-	3.74 ± 0.45 */-
		La Punta	0.73 ± 0.15	1.54 ± 0.15 -/*	3.64 ± 0.91 */-	1.55 ± 0.37 */-
	Phenol	Carrizales	0.45 ± 0.02	0.7 ± 0.12 */-	0.77 ± 0.03 */-	0.72 ± 0.02 */-
		La Punta	0.35 ± 0.07	0.36 ± 0.12	0.71 ± 0.05 */-	0.66 ± 0.09
	2-Phenylethanol	Carrizales	16.25 ± 0.73	35.73 ± 4.29 */*	35.61 ± 0.49 */-	40.19 ± 0.05 */-
		La Punta	17.07 ± 0.62	19.68 ± 1.39 -/*	39.3 ± 3.38 */-	32.82 ± 3.96 */-
	1-Hexanol	Carrizales	0.06 ± 0.06 -/*	0.96 ± 0.2 */*	0.7 ± 0.24	0.79 ± 0
		La Punta	0.3 ± 0.02 -/*	0.48 ± 0.07 -/*	1.04 ± 0.09 */-	0.77 ± 0.06 */-
Aldehydes (ng g^–1^)	Hexanal	Carrizales	59.26 ± 7.24	143.36 ± 2.71 -/*	307.7 ± 49.37 */*	181.88 ± 11.95 */*
		La Punta	48.8 ± 3.19	64.24 ± 7.13 -/*	144.41 ± 15.55 */*	86.62 ± 11.01 */*
	Heptanal	Carrizales	n.d.	n.d.	n.d.	n.d.
		La Punta	n.d.	0.25 ± 0.25	0.39 ± 0.39	n.d.
	(E)-2-Heptenal	Carrizales	4.08 ± 0.23	5.45 ± 0.68	4.53 ± 0.48	9.72 ± 0.19 */-
		La Punta	4.25 ± 0.32	4.9 ± 0.28	7.19 ± 0.99 */-	7.93 ± 1.48 */-
	(E,E)-2,4-Heptadienal	Carrizales	5.14 ± 0.24 -/*	8.77 ± 1.57	7.01 ± 0.78	12.41 ± 1.6 */-
		La Punta	7.64 ± 0.75 -/*	10.97 ± 0.63	10.37 ± 1.95	14.12 ± 2.73
	(E)-2-Octenal	Carrizales	2.06 ± 0.25	3.04 ± 0.08	2.18 ± 0.73	4.18 ± 0.08
		La Punta	1.92 ± 0.39	1.15 ± 0.67	3.2 ± 0.4	3.79 ± 0.77
	Nonanal	Carrizales	13.41 ± 2.01 -/*	20.69 ± 2.1	29.46 ± 8.33	33.25 ± 5.79 */-
		La Punta	9.18 ± 0.74 -/*	15.53 ± 4.44	34.33 ± 7.29 */-	34.45 ± 10.93 */-
	(Z)-6-Nonenal	Carrizales	1.32 ± 0.76 -/*	7.86 ± 0.56	8.41 ± 2.09 */*	8.35 ± 1.88
		La Punta	4.19 ± 0.22 -/*	7.41 ± 1.97	19.53 ± 3.25 */*	10.39 ± 3.46
	(E)-2-Nonenal	Carrizales	161.04 ± 18.39	280.49 ± 33.7 */-	256.82 ± 23.26 */-	378.16 ± 31.09 */-
		La Punta	118.61 ± 11.85	161.24 ± 26.36	378.47 ± 49.02 */-	347.3 ± 34.55 */-
	(E,E)-2,4-Nonadienal	Carrizales	0.64 ± 0.04	1.42 ± 0.22 */-	0.35 ± 0.2	1.15 ± 0.17
		La Punta	0.78 ± 0.04	1.05 ± 0.13	0.94 ± 0.19	1.46 ± 0.37
	(E,Z)-2,6-Nonadienal	Carrizales	449.04 ± 29.3	587.54 ± 85.99	638.66 ± 60.88 */-	1049.28 ± 7.64 */-
		La Punta	356.12 ± 40.47	495 ± 95.57	1053.79 ± 110.05 */-	1059.9 ± 73.41 */-
	(E,E)-2,4-Decadienal	Carrizales	0.5 ± 0.17	0.73 ± 0.12 -/*	0.22 ± 0.22	1.24 ± 0.66
		La Punta	1.07 ± 0.28	2.12 ± 0.24 -/*	1.39 ± 0.63	2.74 ± 1.08
	Benzaldehyde	Carrizales	24.95 ± 1.88	77.57 ± 7.59 */-	67.14 ± 14.86 */-	157.26 ± 6.95 */-
		La Punta	48.07 ± 10.45	46.69 ± 12.01	54.78 ± 8.29	59.07 ± 2.37
	Phenylacetaldehyde	Carrizales	0.74 ± 0.47	15.95 ± 4.17 */*	3.83 ± 0.29	11.76 ± 0.58 */*
		La Punta	2.23 ± 0.45	4.74 ± 0.73 -/*	7 ± 1.54 */-	4.34 ± 1.89 -/*
Esters (ng g^–1^)	2-Methyl propyl acetate	Carrizales	n.d.	0.58 ± 0.58	1.25 ± 0.08	0.93 ± 0.08
		La Punta	n.d.	n.d.	1.02 ± 0.21 */-	1.11 ± 0.24 */-
	(E,E)-2,4-Hexadienoic acid, ethyl ester	Carrizales	1.34 ± 0.15	2.03 ± 0.45 */-	1.76 ± 0.13 -/*	2.98 ± 0.15 */-
		La Punta	1.22 ± 0.1	1.66 ± 0.36	3.53 ± 0.32 */*	3.06 ± 0.22 */-
	Butyl butyrate	Carrizales	0.38 ± 0.22	1.23 ± 0.17 */*	1.03 ± 0.09 */-	1.07 ± 0.02
		La Punta	n.d.	n.d. -/*	1.08 ± 0.09 */-	0.92 ± 0.05 */-
	Ethyl butanoate	Carrizales	4.11 ± 0.23	7.24 ± 1.09 */*	8.18 ± 0.36 */-	8.05 ± 0.44 */-
		La Punta	3.67 ± 0.09	4.21 ± 0.27 -/*	8.29 ± 0.61 */-	7.07 ± 1.08 */-
Apocarotenoids (ng g^–1^)	6-Methyl-5-Hepten-2-one	Carrizales	n.d.	n.d.	0.19 ± 0.19	n.d.
		La Punta	n.d.	n.d.	n.d.	n.d.
	Geranylacetone	Carrizales	n.d.	n.d.	n.d.	0.58 ± 0.58
		La Punta	0.14 ± 0.14	n.d.	n.d.	n.d.
	Beta-ionone	Carrizales	1.59 ± 0.12	2.05 ± 0.29	0.85 ± 0.5	3.26 ± 0.46
		La Punta	1.56 ± 0.11	2.14 ± 0.19	1 ± 0.64	2.38 ± 0.55
	Beta-cyclocitral	Carrizales	n.d.	n.d.	n.d.	0.76 ± 0.76
		La Punta	n.d.	n.d.	n.d.	n.d.

Values followed by a (*/-) show significant differences compared to the non-grafted control (Dunnett’s test; P ≤ 0.05). Values followed by a (-/*) show significant differences compared between the two sites for the same treatment (P ≤ 0.05). Values followed by (*/*) indicate a significant difference compared to the non-grafted control and between the two sites (Dunnett’s test; P ≤ 0.05). Averages were calculated from the biological replicates used in the sensory evaluations*. *From those treatments included in the five sensory evaluations of 2019, NG, and F1Pat81, averages were calculated from six and four biological replicates, respectively, from the three sensory evaluations of La Punta and the two sensory evaluations of Carrizales. Four and three biological replicates were averaged, respectively, for the Shintoza treatment, included in two sensory evaluations from La Punta and one sensory evaluation from Carrizales. Two biological replicates were averaged for the Fian treatment from each of the two sensory evaluations in which this treatment was included (one from La Punta and one from Carrizales).

A significant impact of grafting on the total VOCs was observed in both fields (Figure 9), with the *Cucumis* rootstocks being those that produced fruits with higher VOC content. The fruits produced in plants grafted onto these two rootstocks had VOC profiles with more aldehydes, more alcohols, and more esters. The *Cucurbita* rootstock also increased volatile content, especially alcohols and aldehydes, although this increase was not so important as that of *Cucumis* rootstocks and was only significant in Carrizales.

**FIGURE 10:**
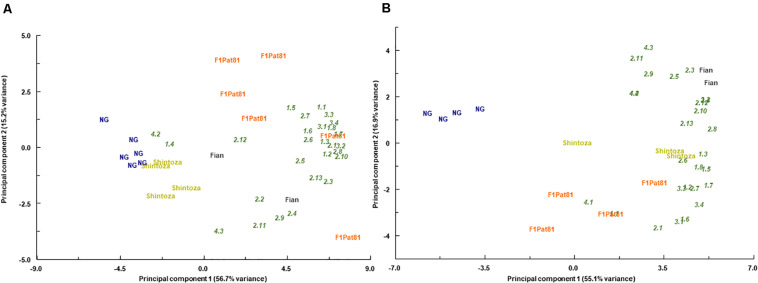
Biplots of scores (bold) and loadings (italics) obtained in the principal component analyses performed with the contents of volatile organic compounds in fruits from non-grafted (NG) and grafted (Shintoza, F_1_Pat81, and Fian) grown at La Punta **(A)** and Carrizales **(B)**. Samples obtained from the sensory analyses performed in 2019 as described in legend of Figure 8. 1_1, 1-pentanol; 1_2, (Z)-3-hexen-1-ol; 1_3, 1-nonanol; 1_4, (Z)-3-nonen-1-ol; 1_5, benzyl alcohol; 1_6, phenol; 1_7, phenylethanol; 1_8, 1-hexanol; 2_1, hexanal; 2_2, heptanal; 2_3, (E)-2-heptenal; 2_4, (E,E)-2,4-heptadienal; 2_5, (E)-2-octenal; 2_6, nonanal; 2_7, (Z)-6-nonenal; 2_8, (E)-2-nonenal; 2_9, (E,E)-2,4-nonadienal; 2_10, (E,Z)-2,6-nonadienal; 2_11, (E,E)-2,4-decadienal; 2_12, benzaldehyde; 2_13, phenylacetaldehyde; 3_1, 2-methyl propyl acetate; 3_2, (E,E)-2,4-headienoic acid, ethyl ester; 3_3, butyl butyrate; 3_4, ethyl butanoate; 4_1, 6-methyl-5-hepten-2-one; 4_2, geranylacetone; 4_3, beta-ionone; 4_4, beta-cyclocitral.

Principal component analyses showed that the different fruit samples were grouped according to their VOC profiles in both fields (Figure 10). Consistently, the samples from NG plants were grouped apart from those obtained from grafted plants, especially those grafted onto *Cucumis*. They were separated according to the first component that explained more than the 50% of the observed variation. This effect was observed in both fields, and fruits grafted onto *Cucumis* rootstocks, both the melon F_1_Pat81 and the wild Fian, resulted in fruits richer in most volatile compounds. This was also true for fruits produced by plants grafted onto the *Cucurbita* rootstock, but the effect of this rootstock was dependent on the field assay, having a similar effect to that of the F_1_Pat81 in Carrizales, but with less impact on plants grown in La Punta. The second component, which explained 15% of the observed variation, separated fruits produced in F_1_Pat81 grafted plants from those produced on Fian grafted plants, with the latter having higher contents in some aldehydes and apocarotenoids (Figure 10).

When analyzed in detail (Table 4), we observed in both fields that the F_1_Pat81 rootstock increased the content of some of the most abundant aldehydes, (E,Z)-2-6-nonadienal, E-2-nonenal, hexanal, and benzaldehyde (the latter more in Carrizales than in La Punta), of most alcohols, with the higher increase occurring in 2-phenylethanol, but also in (Z)-3-hexen-1-ol, 1-nonanol, and benzyl alcohol, and also affected ester content, doubling the amount of ethyl butanoate, compared to the NG melons. Most of these effects were also observed in fruits produced by plants grafted onto the wild melon rootstock (Fian), with some differences, the latter with a higher increase in most aldehydes, except from hexanal.

The analysis of specific products also showed the effect of the *Cucurbita* rootstock, Shintoza. Conversely to the consistent effect found in F_1_Pat81 and Fian, the *Cucurbita* effect was affected by the field, with the impact on VOCs being higher in Carrizales. The impact on aldehydes was less important, with a significant increase in some compounds regarding NG, but lower than that observed in the *Cucumis* rootstocks, for example, for main aldehydes such as (E,Z)-2-6-nonadienal and hexanal, which did not significantly increase in any of the fields. Benzaldehyde and phenylacetaldehyde, however, significantly increased in the saline conditions of Carrizales, even more than in the F_1_Pat81. A similar situation was found for the increase in alcohols and ethyl butanoate, which were only significant in Carrizales.

**FIGURE 11:**
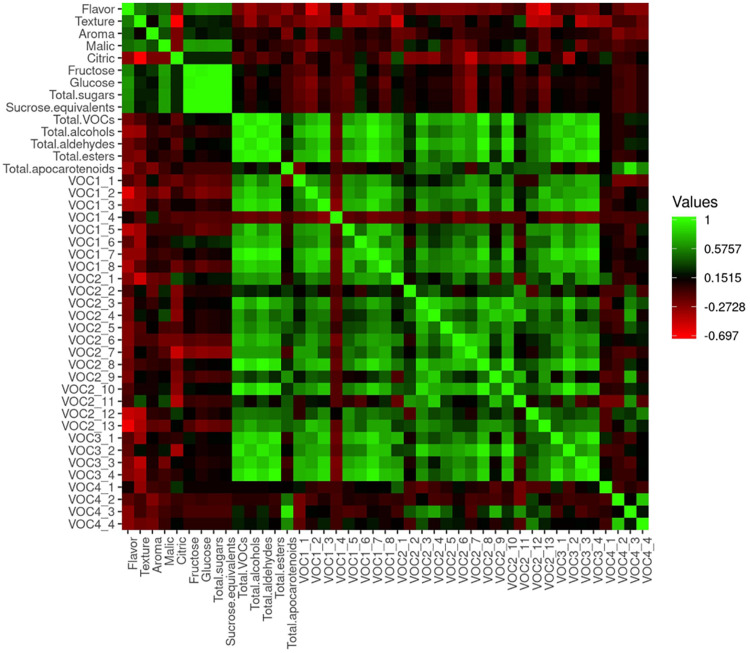
Heatmap of correlation analyses performed with data from the sensory evaluation and metabolite contents of the samples of 2019. 1_1, 1-pentanol; 1_2, (Z)-3-hexen-1-ol; 1_3, 1-nonanol; 1_4, (Z)-3-nonen-1-ol; 1_5, benzyl alcohol; 1_6, phenol; 1_7, 2-phenylethanol; 1_8, 1-hexanol; 2_1, hexanal; 2_2, heptanal; 2_3, (E)-2-heptenal; 2_4, (E,E)-2,4-heptadienal; 2_5, (E)-2-octenal; 2_6, nonanal; 2_7, (Z)-6-nonenal; 2_8, (E)-2-nonenal; 2_9, (E,E)-2,4-nonadienal; 2_10, (E,Z)-2,6-nonadienal; 2_11, (E,E)-2,4-decadienal; 2_12, benzaldehyde; 2_13, phenylacetaldehyde; 3_1, 2-methyl propyl acetate; 3_2, (E,E)-2,4-hexadienoic acid, ethyl ester; 3_3, butyl butyrate; 3_4, ethyl butanoate; 4_1, 6-methyl-5-hepten-2-one; 4_2, geranylacetone; 4_3, beta-ionone; 4_4, beta-cyclocitral.

Correlation analysis performed with sensory scores and metabolite contents is shown in Figure 11. We found moderately significant correlations between flavor and aroma (*R* = 0.42) and between flavor and texture (*R* = 0.53). Flavor acceptability was positively correlated to the sugars (*R* = 0.56 to *R* = 0.63) and malic acid contents (*R* = 0.47), with a higher correlation being detected between flavor and glucose (*R* = 0.63). Significant negative correlations were found between flavor acceptability and contents of (Z)-3-hexen-1-ol (*R* = −0.59), benzyl alcohol (*R* = −0.43), 1-hexanol (*R* = −0.40), benzaldehyde (*R* = −0.50), and phenylacetaldehyde (*R* = −0.64).

## Discussion

Melon production is limited by viral and fungal diseases (García-Jiménez et al., 2008; Lecoq and Katis, 2014; González et al., 2020a). Resistances to the main viruses affecting melons have been reported and introgressed into commercial cultivars. However, traditional varieties, such as snake melons, have been neglected in breeding programs, and most of them are susceptible to most viruses (Solmaz et al., 2016b; Yousif et al., 2007). In the current study, we detected several viruses in different years and fields affecting snake melon plants. The aphid-transmitted *Potyvirus* WMV was the most prevalent, but another potyvirus, ZYMV, and the *Begomovirus* ToLCNDV were also found with a lower incidence. Resistances to all these viruses are available (Sáez et al., 2017; Pérez-de-Castro et al., 2019; Martín-Hernández and Picó, 2021), but have not been introgressed into the snake melon background. Therefore, introgression programs are needed to develop snake melon cultivars with resistance to these viruses.

Regarding the incidence of fungi, global warming is favoring the increasing incidence of highly damaging fungi to melon (Cohen et al., 2012; de Sousa Linhares et al., 2020; Timmusk et al., 2020). *M. phaseolina* and *N. falciformis* were identified as major pathogens in snake melon NG plants. Our inoculation studies confirm the worse response of snake melon, compared to Piel de Sapo sweet melon, to both pathogens, being highly susceptible to *M. phaseolina* and MR to *N. falciformis*. The former has been described as a main pathogen of melon worldwide, with recently available resistant sources (Ambrósio et al., 2015; Cohen et al., 2016; de Sousa Linhares et al., 2020) and the determination of the genetic control under study. The latter has been very recently reported for the first time as a melon pathogen in Spain (González et al., 2020b, c). The high susceptibility of snake melon to Brazilian isolates of *M. phaseolina* compared to sweet melons was already reported by Ambrósio et al. (2015). The response of this melon to *N. falciformis* is reported here for the first time.

Despite the fact that plant mortality caused by these pathogens was moderate, the impact on the production was very important. The dramatic impact of diseases on snake melon production under organic farming management, almost halving yield, was confirmed in the experiment of 2018, when the production of snake melon in infested soils with previous melon cultivation (La Punta) was compared to that obtained in a soil with no previous cultivation of melon (Moncada). Despite production losses, there was not an important effect on fruit traits, other than reduction in FF. Compared to the impact of fungal attack, salts stress of Carrizales in 2018 was less damaging for snake melon plants, and productions similar to those of the unstressed control field were obtained. Therefore, snake melons seem to be much more susceptible to fungal stress than to salinity. This is consistent with the general idea that melons, like other Cucurbits, are only moderately sensitive to salinity, compared to other vegetables (Villalobos et al., 2016; Wang et al., 2016). As occurred with fungal stress, FF was slightly reduced under salt stress, which also increases SSC. These effects have been previously reported in sweet melons with higher SSC when grown at high salinity levels (Colla et al., 2006), but our results also show a similar effect even in this non-sweet melon. Also, an effect of salinity on fruit firmness has been reported (Trajkova et al., 2006; Yarsi et al., 2012).

Grafting can be used to reduce the impact of stressful conditions on snake melon production and quality. In fact, the use of *Cucumis* rootstocks, both melon, F_1_Pat81, and wild *Cucumis*, Fian and Fimy, reduced plant mortality. *M. phaseolina*, *M. cannonballus*, and different species of *Fusarium* were detected in plants cultivated in both fields. Previous studies showed that F_1_Pat81, Fian, and Fimy are quite tolerant to *M. cannonballus* and *M. phaseolina* (Ambrósio et al., 2015; Cáceres et al., 2017; Gisbert et al., 2020; Castro et al., 2020), although the tolerance derived from Pat 81 seems to be temperature dependent (Castro et al., 2020; de Sousa Linhares et al., 2020). The wild *Cucumis* are also resistant to *F. oxysporum* (Matsumoto et al., 2011; Gisbert et al., 2020). These tolerances may account for the reduced mortality found in snake melon plants grafted onto these rootstocks. *Cucurbita* rootstocks are known to have resistance to *F. oxysporum* and tolerance to *M. cannonballus* and *M. phaseolina*. However, in our study, plant mortality similar to that found on NG snake melon occurred in *Cucurbita* grafted plants. Regarding the lower performance of *Cucurbita* rootstocks, it should be considered that these are usually employed to control *F. oxysporum.* This fungus, along with *M. phaseolina* and *M. cannonballus*, were detected in *Cucurbita* roots. However, in this case, the high levels of mortality could be due to the presence of other fungi as *N. falciformis* and *N. keratoplastica* of the FSSC (O’Donnell et al., 2008; González et al., 2020a), as *F. solani* has been found to very seriously affect the *Cucurbita* hybrid rootstocks in watermelon (Armengol et al., 2000). It is then necessary to include these new pathogens in the selection process of new rootstocks for melon to overcome future pathogenic problems in these *Cucurbita* species, due to the possible spread of these soilborne fungi in our producing areas.

As stated before, grafting onto the melon F_1_Pat81 and wild *Cucumis* rootstocks reduced consistently plant mortality in different agroecological conditions, but the impact on the production per plant was variable between years and fields, and similar to that caused by the *Cucurbita* rootstocks. Despite the variability, in most cases, grafted plants displayed lower production losses than NG, except those grafted onto the Fimy rootstock, which were less productive. Under salt stress, grafting significantly increased production per plant, as occurred in 2018 in Carrizales, with similar effect of melon and *Cucurbita* rootstocks. The combined effect of salinity and fungal stress, and the environmental conditions that favored the stressful scenario, caused the highest impact on production in Carrizales in 2019. Increased susceptibility to soil borne diseases has been reported in tomato under high salinity (Bai et al., 2018). Also, in melons, enhanced fungal damage has been reported under saline conditions (Roustaee et al., 2012; Mirtalebi and Banihashemi, 2019). Grafting contributed to alleviate the impact of these extreme conditions. The lower performance of F_1_Pat81 in these conditions could be related to the higher temperatures, as the level of resistance against *M. phaseolina* and *M. cannonballus* of materials derived from F_1_Pat81 drop at higher temperatures (Castro et al., 2020; de Sousa Linhares et al., 2020). Therefore, grafting is a good strategy to reduce plant mortality due to fungal stress and can alleviate yield losses, depending on the resistance of the rootstocks and the fungal profile of the soil, and increase production under saline conditions.

The use of grafting in melon is prevented by the impact that the different rootstocks can have on fruit quality (Fita et al., 2007; Fallik and Ilic, 2014; Fredes et al., 2017; Leonardi et al., 2017). In the case of snake melons, the effect of the different rootstocks on fruit traits, such as shape, firmness, pH, or Brix degree, was minimum for both *Cucumis* and *Cucurbita* rootstocks, and only limited effects in certain combinations and environments were detected. Most of these mild effects were associated to the flesh and rind color. Variable effects on fruit color have also been reported for different types of rootstocks in sweet melons (Cáceres et al., 2017). The increase in Brix degree observed in NG snake melons cultivated under salt pressure in Carrizales was also consistently observed both years in grafted plants, which is more intense in plants grafted onto *Cucumis* rootstocks.

Even when no effect of rootstocks on basic fruit traits is detected, grafting can alter metabolite profiles, which may affect consumers’ preferences. Trained sensory panels have been successfully used for the evaluation of sweet melons (Bianchi et al., 2016; Park et al., 2018; Ayres et al., 2019), Spanish sweet melons (traditional landraces) (Escribano and Lázaro, 2012), and even snake melons (Omari et al., 2018). Training such panels is time-consuming and much more difficult to implement in neglected crops such as snake melon. Despite these difficulties, in this study, they consistently showed preferences for snake melon fruits produced in NG plants. In this sense, *Cucurbita* hybrid rootstocks clearly scored below the NG control in most sessions, while the *Cucumis* rootstock F_1_Pat81 showed no significant differences with the NG control. It is also important to note that some tasters reported fibrous or course textures and strange tastes for the *Cucurbita* grafted plant fruits.

We performed analysis of sugars, acids, and volatiles to evaluate if these consumer preferences might be associated to metabolic profile differences due to grafting. Snake melons do not accumulate sucrose, or present it at very low levels. Burger et al. (2003) related this trait with a high acid invertase activity that prevents the accumulation of sucrose and described the recessive gene *suc* as responsible for high sugar accumulation in sweet melons. The fruits analyzed in this work have shown this profile, with sucrose remaining under the limits of quantification and limited accumulation of hexoses. The main acids detected in our snake melon fruits were citric and malic, with malic being predominant. Burger et al. (2003) described the same profile in the variety “Faqqous” of snake melon, with high acid values being conferred by the dominant gene *So*. Snake melons (along with Indian Momordica and Acidulus melons) are considered sour melons and, in contrast to sweet melons, have malic acid as the main organic acid. The non-sweet and acidic profile found in our snake melon is typical of the *So/So*, *Suc/Suc* combination, described in other *C. melo* var. *flexuosus* varieties (Burger et al., 2003). Cohen et al. (2014) identified the gene *CmPH* with a major effect on fruit acidity. They found a high acid profile in the cultivars that lacked a four-amino acid duplication in this gene, among them the *flexuosus* melons.

Grafting seems to affect the sugars and organic acid profile, with this effect being more accentuated with *Cucurbita* rootstocks that significantly decrease both hexoses, affecting sucrose equivalents and malic acid. Melon rootstocks did not alter malic acid content, and the effect on sugars was only significant for glucose, not affecting sucrose equivalents. The sugar and organic acid contents can affect consumer acceptability. In fact, flavor scores of sensory evaluations were positively correlated with sugars and malic acid contents.

Snake melons are climacteric melons, aromatic when fully mature (Esteras et al., 2018), but we characterized the aroma profile at commercial maturity, when fruits are physiologically immature. At this ripening stage, VOC profile was more like that of non-climacteric melons, very rich in aldehydes, followed at a considerable distance by alcohols, and low levels of esters and apocarotenoids. The aldehyde profile of the snake melon was characterized by high contents of (E,Z)-2-6-nonadienal, followed by E-2-nonenal, hexanal, and benzaldehyde, which differ from the aldehyde profile usually found on aromatic and non-aromatic sweet melons. These compounds are known to be some of those with the main impact in melon aroma (Gonda et al., 2016). (E,Z)-2-6-nonadienal is reported to contribute with cucumber-like odor, whereas E-2-nonenal and hexanal contribute with green and fresh odor notes. This profile differs from that of sweet climacteric melons, poorer in these aldehydes, but also from other non-climacteric melons, such as the inodorus group, rich in aldehydes, but with a different aldehyde profile, richer in hexanal (Esteras et al., 2018). The alcohol profile was also different to that of aromatic sweet melons, richer in hexanol, stale odor, and fermented notes, and Z-3-hexen–1-ol, grassy-green odor, instead of 2-phenylethanol, floral odor, the main alcohol present in snake melons. The ester compounds, which are the major contributors to the aroma of sweet melons, are nearly absent in this immature fruit, with ethyl butanoate being a major ester contributor to melon aroma and the most abundant, although in very low amounts compared to sweet melons. Also, the apocarotenoid beta-ionone was the only consistently detected, but in very low amounts. This profile was similar to that reported for the “Cai Gua” snake melon by Tang et al. (2015) and Chen et al. (2016), who also reported high levels of phenylethanol among alcohols, and (E-Z)-2-6-nonedienal, (E)-2-nonenal, hexanal, and benzaldehyde among aldehydes.

There was a clear impact of grafting on VOC profile, with a higher effect in *Cucumis* vs *Cucurbita* rootstocks. Grafting onto *Cucumis* rootstocks resulted in fruits with a VOC profile richer in most aldehydes, alcohols, and esters. This effect was lower and more dependent on the field conditions in *Cucurbita* rootstocks. Flavor acceptability showed significant negative correlations with some of these compounds, (Z)-3-hexen-1-ol, benzyl alcohol, and 1-hexanol, among alcohols, and phenylacetaldehyde and benzaldehyde, among aldehydes. All grafted plants had significant increases of most of these compounds compared to NG plants. The increase was higher for *Cucurbita* vs *Cucumis* rootstocks in Carrizales for (Z)-3-hexen-1-ol, the compound with the highest negative correlation with flavor acceptability. The increase in the two aldehydes more negatively correlated with flavor acceptability, phenylacetaldehyde and benzaldehyde, was more important in *Cucurbita* in the Carrizales assay, associated with the lower scoring of fruits produced by plants grafted onto these rootstocks in the sensory evaluations.

Our results showed that *Cucurbita* rootstock has a higher impact in sugar and organic acid profile than in VOC profile, resulting in a less favorable consumer perception, and that *Cucumis* rootstocks affect VOC profile more than sugar and acid profile, which may result in a lower effect on consumer perception, although the increase in specific alcohols and aldehydes could also be related to the less positive perception of consumers of fruits coming from grafted plants.

## Conclusion

Snake melon seems to be moderately susceptible to biotic stress and especially to soilborne diseases. Nonetheless, under high incidence conditions, yield losses can be higher than 50%. This yield loss would be even higher when combined with high salinity, due to a synergic effect. In organic farming, strategies against diseases are limited, and in this context, the use of rootstocks seems to be an efficient alternative. The use of *Cucumis* rootstocks seems to be more favorable. F_1_Pat81 not only reduced yield losses under biotic stress but also increased yield under salt stress. Nonetheless, grafting may have a side effect on consumer acceptability. This effect seems to be related to changes in the profiles of the analyzed metabolites caused by grafting, but it depends on the specific scion–rootstock combination. *Cucumis* rootstocks had a major effect on the VOC profile, but the incidence on sugar accumulation was limited compared to *Cucurbita* rootstocks, probably reducing the negative side impact on flavor.

## Data Availability Statement

The original contributions presented in the study are included in the article/Supplementary Material, further inquiries can be directed to the corresponding author/s.

## Author Contributions

BP, JC-C, SG-M, JR, and AF-L conceived and designed the research. AF-L, SG-M, and JR managed field assays, fruit characterization, and sampling with contributions from AS, MF, CL, CG, and BP. BP, CG, and AF-L performed the sensory evaluations. MD contributed to the selection of the snake melon landraces. CG contributed to the selection of the rootstocks. VG, AG-C, and CJ performed fungal isolations and identification. CL, AP-D-C, and AS contributed to the analysis of virus. JC-C and RM performed the metabolite analysis. AF-L, JC-C, and BP wrote the manuscript with important contributions from CG, AP-D-C, VG, and AG-C. All authors critically read and approved the final manuscript.

## Conflict of Interest

The authors declare that the research was conducted in the absence of any commercial or financial relationships that could be construed as a potential conflict of interest.

## Footnotes

1 www.aemet.es

2 http://riegos.ivia.es/red-siar

3 http://www.westerdijkinstitute.nl/fusarium/

4 http://www.heatmapper.ca

## Supplementary Material

Supplementary Figure 1 Three fields used for melon cultivation: Moncada (Valencia) (A), La Punta (Valencia) **(B)**, and Carrizales (Alicante) **(C)**.

Supplementary Figure 2 Rootstock-scion union of snake melon plants grafted onto different rootstocks, F1Pat81 **(A)**, Fian **(B)**, Fimy **(C)**, Shintoza **(D)**, Cobalt **(E)**, and non-grafted snake melon **(F)**. All graftings were compatible and resulted in fully developed adult plants. Differences in hypocotyl diameters between scion and rootstocks can be observed, thicker in *Cucurbita* **(D,E)** and thinner in wild *Cucumis* **(B,C)** compared to the melon scion.

Supplementary Figure 3 Mean temperature (lines) and total rainfall (columns) for each field in 2018 **(A)** and 2019 **(B)**.

Supplementary Figure 4 Pests and diseases detected in La Punta. Aphids on a snake melon plant **(A)**. Snake melon plants with WMV **(B)** and ZYMV symptoms **(C)**. Plants affected by *P. xanthii* **(D)**.

Supplementary Figure 5 Snake melon plants affected by different soil borne pathogens in Carrizales (2019), with their corresponding roots. **(A,B)** Non-grafted, **(C,D)** grafted onto Shintoza, **(E,F)** grafted onto Cobalt.

Supplementary Figure 6 Response of snake melon to *Monosporascus cannonballus* (left) and *Macrophomina phaseolina* (right). Healthy root of non-inoculated control **(A)**. Roots of plants inoculated with *M. cannonballus* at 30 DAI, root lesions and pruning are apparent **(B)**. Response of snake melon to *M. phaseolina*. Plant inoculated with the toothpick method **(C)**. Lesions of *M. phaseolina* on the stem at 15 DAI **(D)** and lesion on the stem of a dead plant at 30 DAI **(E)**.

Supplementary Figure 7 Response of snake melon to *N. falciformis* and *N. keratoplastica*. Pictures show the symptoms at 7 and 14 DAI, and the non-inoculated control.

Supplementary Table 1 Volatiles analyzed in the fruit samples. (CAS, Chemical Abstract Service, MW: molecular weight; Chromatographic parameters obtained from GC-MS chromatograms: Rt: retention time, RI: retention index; Quan ion: quantitation ion). Retention index calculated with n-alkanes on Supelcowax 10 (bonded polyethylene glycol) capillary column. Linearity range corresponding to the real concentration of standards used for calibration.
