# Supplementary material for: Grafting Snake Melon [Cucumis melo L. subsp. melo Var. flexuosus (L.) Naudin] in Organic Farming: Effects on Agronomic Performance; Resistance to Pathogens; Sugar, Acid, and VOC Profiles; and Consumer Acceptance
Source: Front Plant Sci. 2021 Feb 19;12:613845. doi: 10.3389/fpls.2021.613845 (PMC7933694; doi:10.3389/fpls.2021.613845)
Supplement: Supplementary Figure 1 — Three fields used for melon cultivation: Moncada (Valencia) (A), La Punta (Valencia) (B), and Carrizales (Alicante) (C). [file Presentation_1.PPTX]

## Slide 1
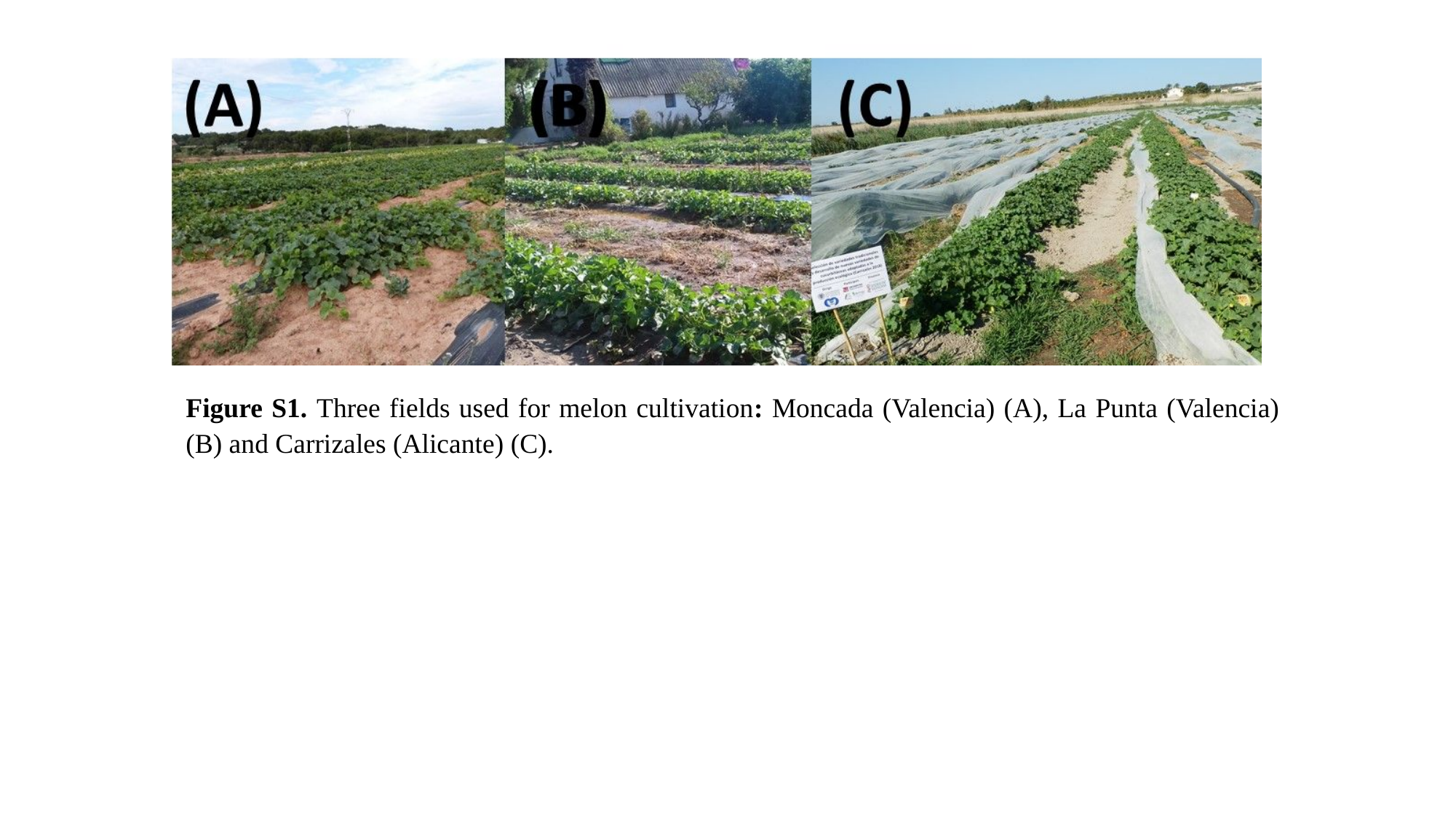

Figure S1. Three fields used for melon cultivation: Moncada (Valencia) (A), La Punta (Valencia) (B) and Carrizales (Alicante) (C).

## Slide 2
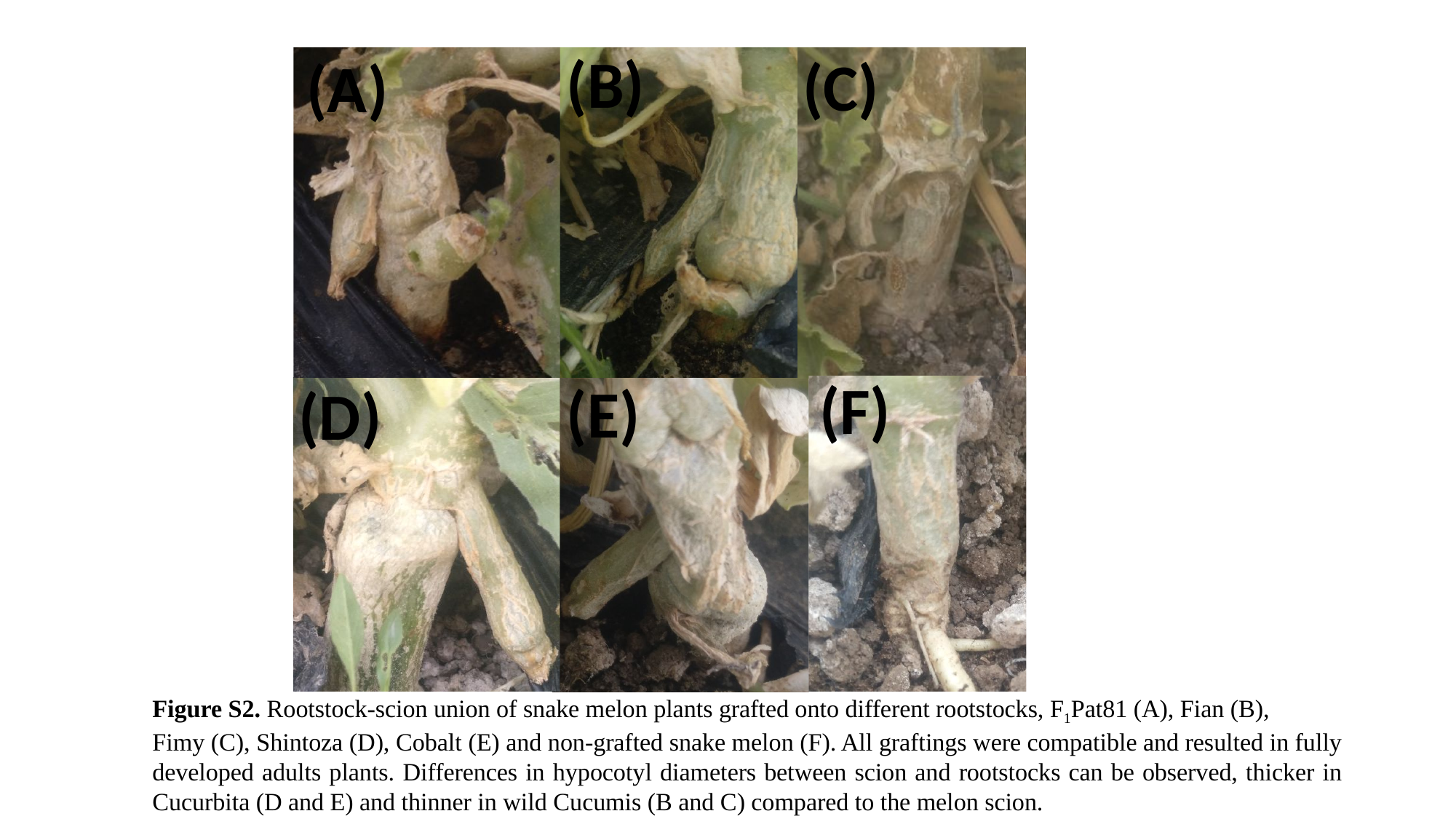

(B)
(C)
(A)
(F)
(E)
(D)
Figure S2. Rootstock-scion union of snake melon plants grafted onto different rootstocks, F1Pat81 (A), Fian (B),
Fimy (C), Shintoza (D), Cobalt (E) and non-grafted snake melon (F). All graftings were compatible and resulted in fully developed adults plants. Differences in hypocotyl diameters between scion and rootstocks can be observed, thicker in Cucurbita (D and E) and thinner in wild Cucumis (B and C) compared to the melon scion.

## Slide 3
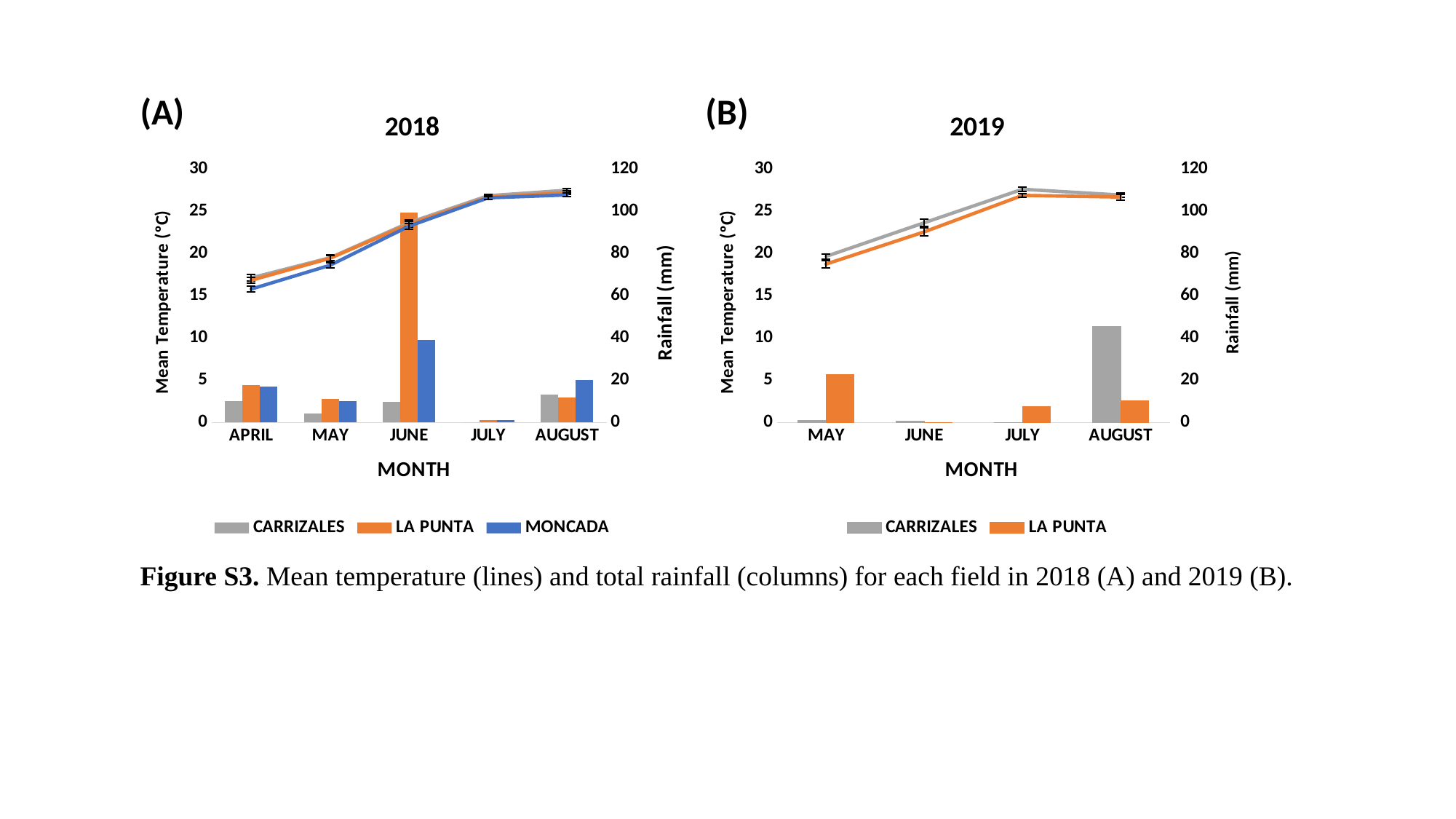

### Chart: 2018
| Category | CARRIZALES | LA PUNTA | MONCADA | CARRIZALES | LA PUNTA | MONCADA |
|---|---|---|---|---|---|---|
### Chart: 2019
| Category | CARRIZALES | LA PUNTA | CARRIZALES | LA PUNTA |
|---|---|---|---|---|Figure S3. Mean temperature (lines) and total rainfall (columns) for each field in 2018 (A) and 2019 (B).

## Slide 4
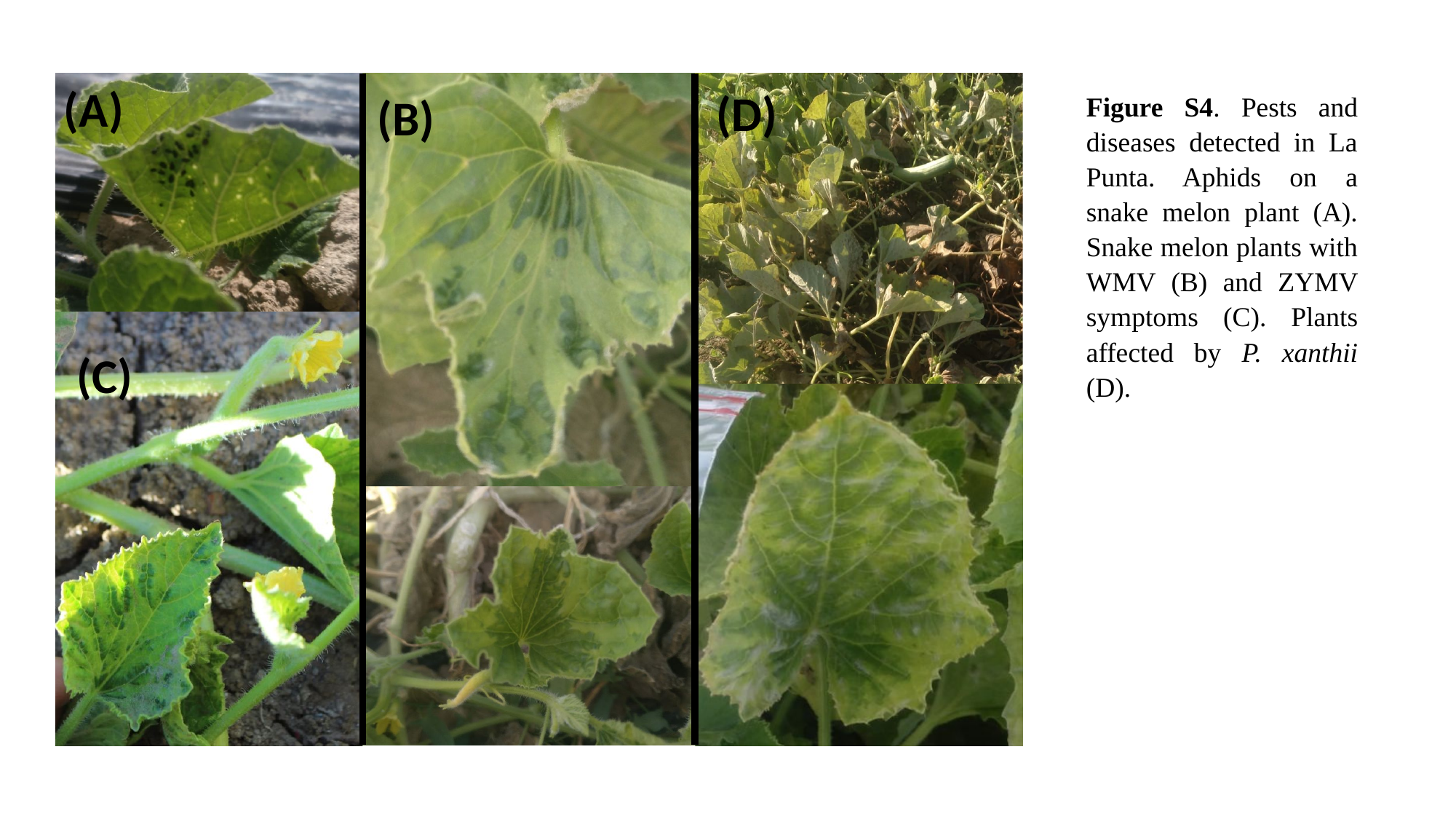

(A)
(D)
(B)
(C)
Figure S4. Pests and diseases detected in La Punta. Aphids on a snake melon plant (A). Snake melon plants with WMV (B) and ZYMV symptoms (C). Plants affected by P. xanthii (D).

## Slide 5
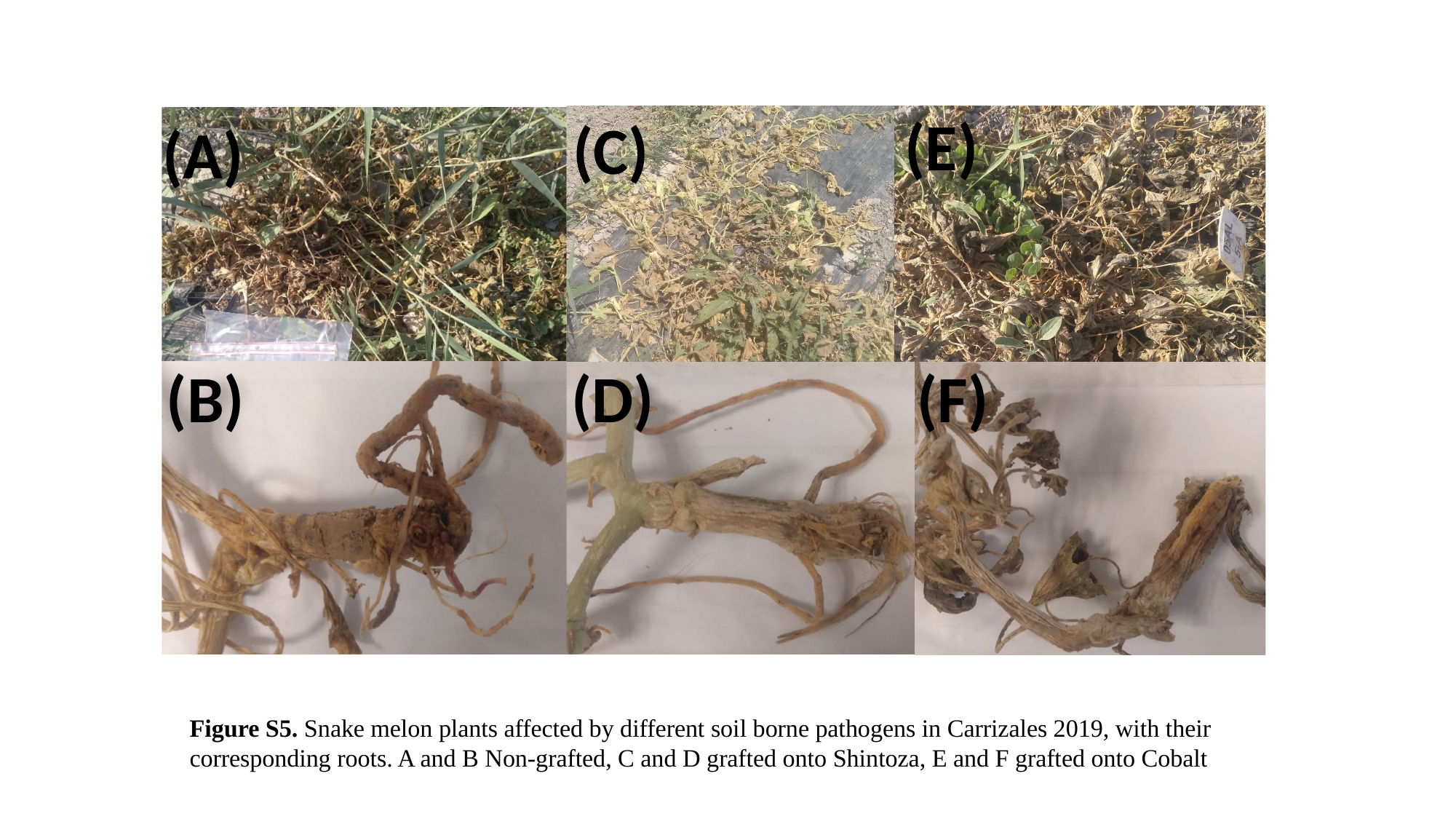

(E)
(C)
(A)
(B)
(D)
(F)
Figure S5. Snake melon plants affected by different soil borne pathogens in Carrizales 2019, with their corresponding roots. A and B Non-grafted, C and D grafted onto Shintoza, E and F grafted onto Cobalt

## Slide 6
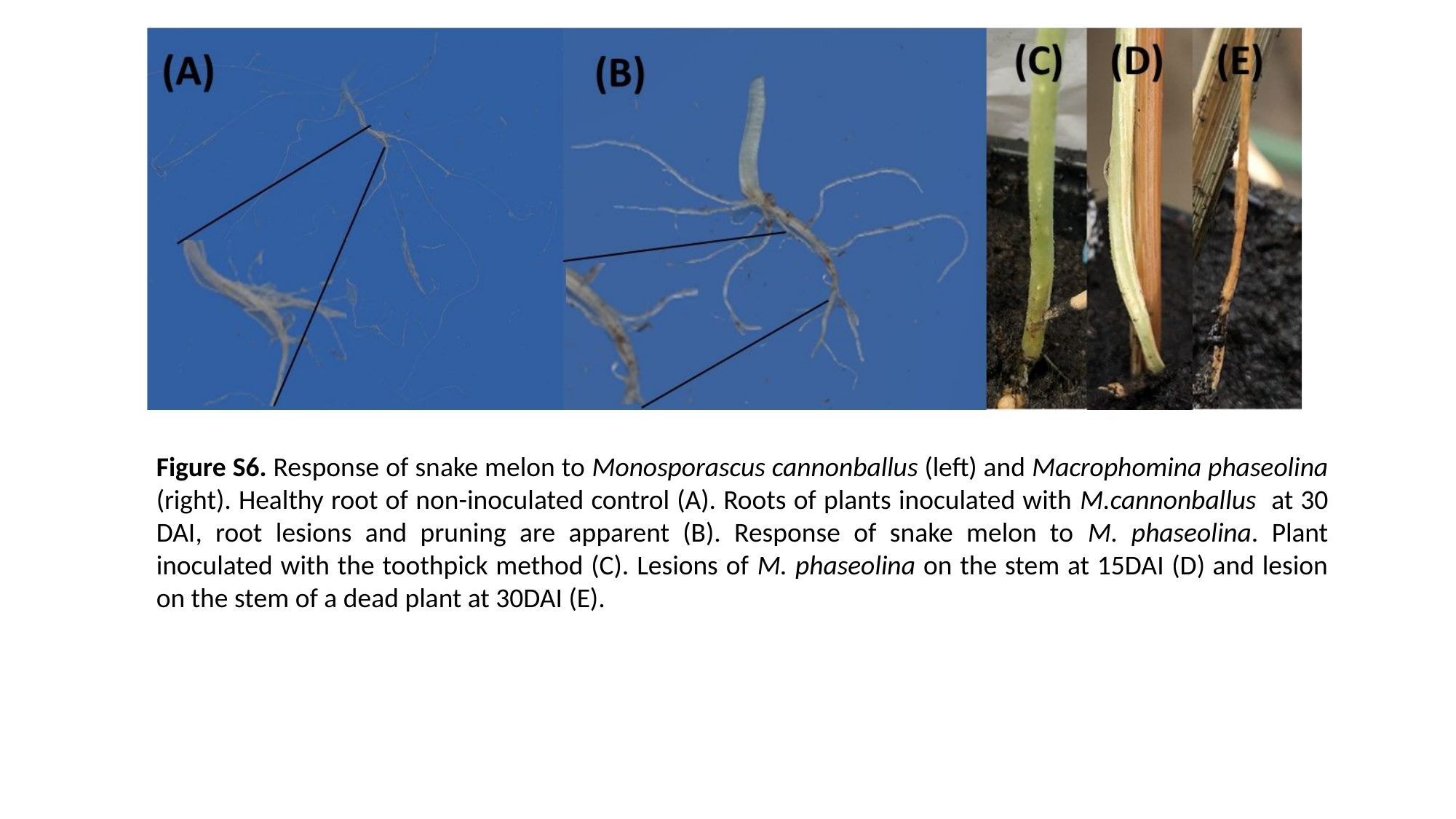

Figure S6. Response of snake melon to Monosporascus cannonballus (left) and Macrophomina phaseolina (right). Healthy root of non-inoculated control (A). Roots of plants inoculated with M.cannonballus at 30 DAI, root lesions and pruning are apparent (B). Response of snake melon to M. phaseolina. Plant inoculated with the toothpick method (C). Lesions of M. phaseolina on the stem at 15DAI (D) and lesion on the stem of a dead plant at 30DAI (E).

## Slide 7
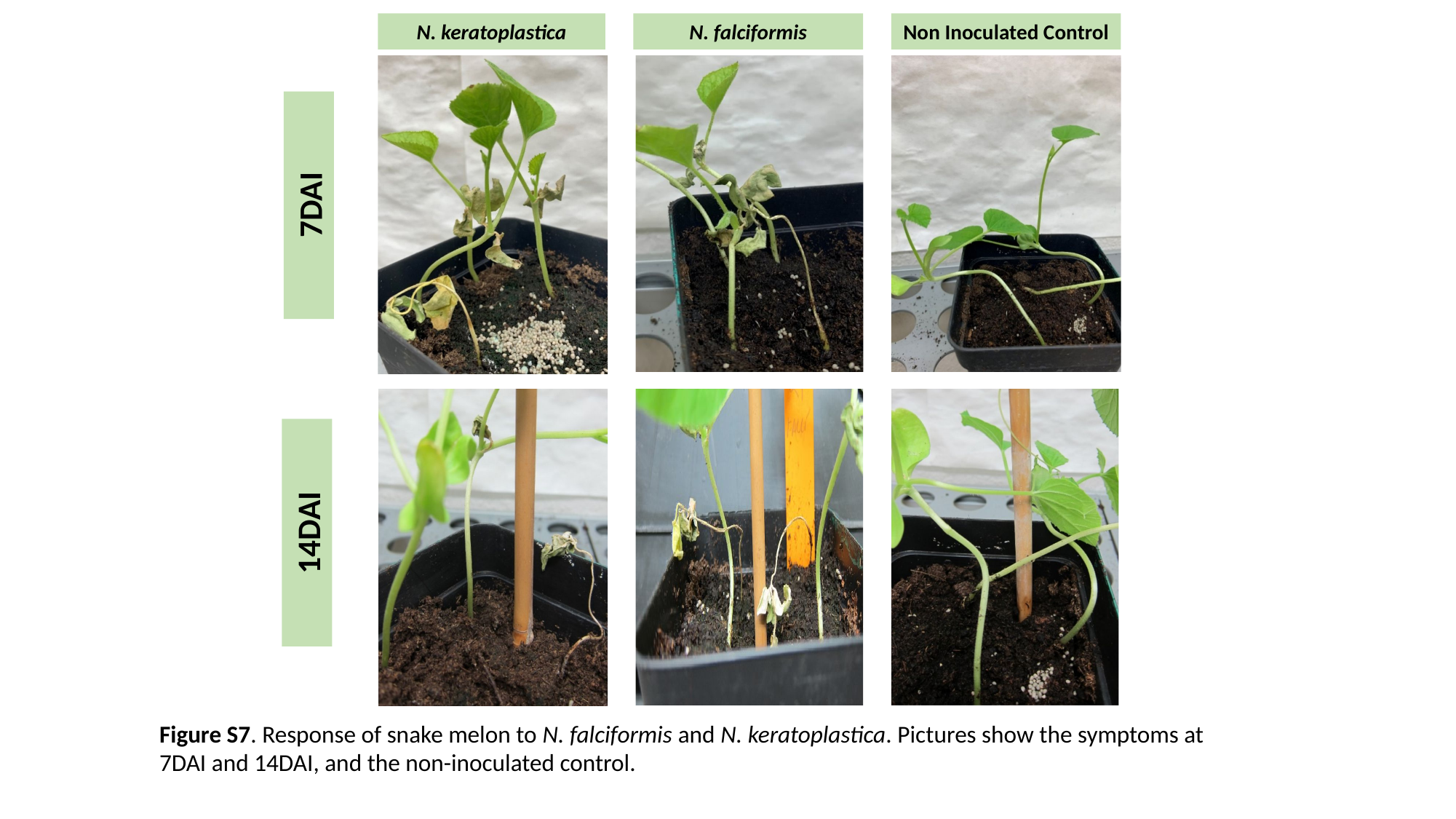

Non Inoculated Control
N. keratoplastica
N. falciformis
7DAI
14DAI
Figure S7. Response of snake melon to N. falciformis and N. keratoplastica. Pictures show the symptoms at 7DAI and 14DAI, and the non-inoculated control.
