# Supplementary material for: Grafting Snake Melon [Cucumis melo L. subsp. melo Var. flexuosus (L.) Naudin] in Organic Farming: Effects on Agronomic Performance; Resistance to Pathogens; Sugar, Acid, and VOC Profiles; and Consumer Acceptance
Source: Front Plant Sci. 2021 Feb 19;12:613845. doi: 10.3389/fpls.2021.613845 (PMC7933694; doi:10.3389/fpls.2021.613845)
Supplement: Supplementary Table 1 — Volatiles analyzed in the fruit samples. (CAS, Chemical Abstract Service, MW: molecular weight; Chromatographic parameters obtained from GC-MS chromatograms: Rt: retention time, RI: retention index; Quan ion: quantitation ion). Retention index calculated with n-alkanes on Supelcowax 10 (bonded polyethylene glycol) capillary column. Linearity range corresponding to the real concentration of standards used for calibration. [file Table_1.docx]

**Supp. Table 1.** Volatiles analyzed in the fruit simples. (CAS, Chemical Abstract Service, MW: molecular weight; Chromatographic parameters obtained from GC-MS chromatograms: Rt: retention time, RI: retention index; Quan ion: quantitation ion). Retention index calculated with n-alkanes on Supelcowax 10 (bonded polyethylene glycol) capillary column. Linearity range corresponding to the real concentration of standards used for calibration.

|  | Compound name | CAS No. | MW | Rt (min) | RI  (Supelcowax 10) | Quan Ion (m/z) | Linear range  (ng mL^-1^) | r^2^ |
| --- | --- | --- | --- | --- | --- | --- | --- | --- |
| Alcohols | 1-Pentanol | 71-41.0 | 88 | 12.91 | 1162 | 55 | 130-2595 | 1 |
|  | 1-hexanol | 111-27-3 | 102 | 16.54 | 1267 | 56 | 26-2568 | 0.999 |
|  | (Z)-3-hexen-1-ol | 928-96-1 | 100 | 17.57 | 1297 | 67 | 64-3212 | 0.999 |
|  | 1-nonanol | 143-08-8 | 144 | 26.15 | 1585 | 55 | 23-2324 | 0.998 |
|  | (Z)-3-nonen-1-ol | 10340-23-5 | 142 | 26.8 | 1600 | 67 | 157-3132 | 0.999 |
|  | (Z)-6-nonen-1-ol | 35854-86-5 | 142 | 27.75 | 1634 | 67 | 30-2958 | 0.998 |
|  | (E,Z)-2,6-nonadien-1-ol | 28069-72-9 | 140 | 29.15 | 1685 | 67 | 34-3425 | 0.997 |
|  | Benzyl alcohol | 100-51-6 | 108 | 31.99 | 1795 | 79 | 57-5721 | 0.999 |
|  | 2-phenylethanol | 60-12-8 | 122 | 32.85 | 1830 | 91 | 35-3542 | 0.998 |
|  | Phenol | 108-95-2 | 94 | 35.24 | 1937 | 94 | 21-2147 | 0.999 |
| Aldehydes | Hexanal | 66-25-1 | 100 | 7.2 | 993 | 56 | 54-2701 | 0.999 |
|  | (E)-2-methyl-2-butenal | 497-03-0 | 84 | 7.32 | 995 | 84 | 175-3499 | 0.999 |
|  | Heptanal | 111-71-7 | 114 | 10.49 | 1096 | 70 | 202-2020 | 0.996 |
|  | (E)-2-heptenal | 18829-55-5 | 112 | 15.48 | 1235 | 83 | 150-3004 | 0.997 |
|  | Nonanal | 124-19-6 | 142 | 17.9 | 1307 | 57 | 54-2713 | 0.999 |
|  | (E)-2-octenal | 2548-87-0 | 126 | 19.05 | 1343 | 55 | 66-3287 | 0.998 |
|  | (Z)-6-nonenal | 2277-19-2 | 140 | 19.75 | 1366 | 81 | 167-3345 | 0.998 |
|  | (E,E)-2,4-heptadienal | 881395 | 110 | 21.17 | 1408 | 81 | 65-3252 | 0.998 |
|  | Benzaldehyde | 110-52-7 | 106 | 21.97 | 1436 | 105 | 45-2268 | 0.999 |
|  | (E)-2-nonenal | 18829-56-6 | 140 | 22.43 | 1450 | 81 | 236-2360 | 0.991 |
|  | (E,Z)-2,6-nonadienal | 557-48-2 | 138 | 23.95 | 1501 | 70 | 64-3205 | 0.999 |
|  | Phenylacetaldehyde | 122-78-1 | 120 | 25.59 | 1559 | 91 | 465-2324 | 0.994 |
|  | (E,E)-2,4-nonadienal | 5910-87-2 | 138 | 27.32 | 1618 | 81 | 71-3557 | 0.997 |
|  | (E,E)-2,4-decadienal | 25152-84-5 | 152 | 30.3 | 1727 | 81 | 56-2804 | 1 |
| Esters | Benzyl acetate | 140-11-4 | 150 | 28.04 | 1646 | 108 | 30-2952 | 0.998 |
|  | 2-Methyl propyl acetate | 110-19-0 | 116 | 4.67 | 926 | 43 | 111-2765 | 1 |
|  | Ethyl butanoate | 105-54-4 | 116 | 5.94 | 947 | 71 | 265-2648 | 1 |
|  | Butyl buryrate | 109-21-7 | 144 | 12.31 | 1131 | 71 | 30 -2965 | 1 |
|  | (E,E)-2,4-hexadienoic acid, ethyl ester | 2396-84-1 | 140 | 21.58 | 1423 | 67 | 33-3303 | 0.999 |
| Apocarotenoids | 6-methyl-5-hepten-2-one | 110-93-0 | 126 | 15.99 | 1251 | 109 | 32-3218 | 0.998 |
|  | Geranylacetone | 689-67-8 | 194 | 31.44 | 1773 | 43 | 32-3174 | 0.998 |
|  | β-ionone | 14901-07-6 | 192 | 33.49 | 1856 | 177 | 34-3390 | 0.998 |
